# Living Biotherapeutics Using Nanoparticles‐Armed Cyanobacteria for Boosting Photodynamic‐Immunotherapy of Cancer

**DOI:** 10.1002/advs.202502746

**Published:** 2025-05-08

**Authors:** Zhengwei Xu, Mingsong Zang, Hui Li, Ruizhen Tian, Zherui Zhang, Wang Liu, Fei Xiao, Xuesha Yan, Yan Zhu, Canhong Zhu, Jiayun Xu, Shuangjiang Yu, Tingting Wang, Hongcheng Sun, Junqiu Liu

**Affiliations:** ^1^ College of Material, Chemistry and Chemical Engineering, Key Laboratory of Organosilicon Chemistry and Material Technology, Ministry of Education Hangzhou Normal University Hangzhou 311121 P. R. China; ^2^ College of Life and Environmental Sciences Hangzhou Normal University Hangzhou 311121 P. R. China

**Keywords:** cancer immunotherapy, covalent self‐assembly, hypoxic tumor, living biotherapeutics, photodynamic immunotherapy

## Abstract

The interdisciplinary development of synthetic biology and material sciences propels medicine into a new era. For cancer therapy, living biotherapeutics integrating functional living bacteria with nanomedicine are particularly interesting. The current study developed a living biotherapeutic platform integrating oxygen‐self‐supplying cyanobacteria with multifunctional prodrug nanoparticles to boost photodynamic immunotherapy. Generally, tetracarboxyl porphyrin is associated with cisplatin via a covalent self‐assembly strategy into uniform prodrug‐skeletal nanoparticles (ZnNCs). This helped encapsulate the antitumor drug dicumarol derivative (DicTBS). Later, these developed DicTBS‐ZnNC nanoparticles helped arm the surface of cyanobacteria using electrostatic adsorption to yield living nanotherapeutics (Cyano@DicTBS‐ZnNCs). Cyano@DicTBS‐ZnNCs achieved a self‐supply of nanoparticles and oxygen under 660 nm laser irradiation, producing PDT therapeutic effects. Furthermore, combining cisplatin and dicoumarol achieved synergistic anticancer effects. This approach also induced immunogenic cell death (ICD) and regulated the tumor microenvironment (TME). This promoted an immune‐supportive environment to improve antitumor immune responses.

## Graphical abstract

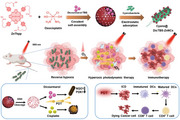

## 1 Introduction

Rapid recovery, noninvasiveness, and spatiotemporal controllability characterize Photodynamic therapy (PDT).^[^ ^1^ ^]^ PDT can directly kill cancer cells and induce immunogenic cell death (ICD) using reactive oxygen species (ROS).^[^ ^2^ ^]^ The primary PDT mechanism involves the excitation of photosensitizers using appropriate light. This is followed by the incomplete reduction of endogenous oxygen, synthesizing cytotoxic ROS.^[^ ^3^, ^4^ ^]^ This method has become the preferred clinical technique for treating superficial tumors.^[^ ^5^ ^]^ Therefore, PDT efficacy depends on the tumor specificity of the photosensitizer, light delivery density, and oxygen content in the tumor tissue. Previous studies have described that PDT effectiveness is limited by oxygen reliance, restricting its application against hypoxic tumors. Hypoxia is a common characteristic of advanced solid tumors because of rapid tumor growth and abnormal tumor vasculature, inducing insufficient oxygen delivery to the tissues.^[^ ^6^, ^7^ ^]^ The O_2_ depletion regulated by the photosensitizer exacerbates tumor tissue hypoxia, inhibiting PDT efficacy.^[^ ^8^ ^]^ Consequently, tumor hypoxia has emerged as a major bottleneck for PDT.^[^ ^9^ ^]^ Recently, increasing research has focused on strategies to elevate the oxygen content in the tumor microenvironment and improve PDT efficacy.

Most PDT systems consume O_2_ to generate ROS. Developing strategies to effectively increase O_2_ concentration in the hypoxic tumor enhances PDT efficacy.^[^ ^10^, ^11^, ^12^ ^]^ For example, carriers such as hemoglobin,^[^ ^13^ ^]^ perfluorocarbons,^[^ ^14^ ^]^ and metal–organic frameworks (MOFs)^[^ ^15^ ^]^ help deliver oxygen to tumor sites, providing additional oxygen for PDT. However, these carriers face uneven distribution, precise control difficulty, low oxygen loading capacity, and poor biocompatibility. Another strategy to improve the O_2_ content in a tumor is in situ catalytic decomposition of endogenous H_2_O_2_ to produce O_2,_ including CAT^[^ ^16^ ^]^ and Nanozymes with CAT‐like activity.^[^ ^17^ ^]^ However, these methods are limited by the low endogenous H_2_O_2_ levels, low catalytic efficiency, and potential metal catalyst toxicity.^[^ ^16^, ^18^ ^]^ An alternative strategy has been proposed, using biomimetic photosynthesis to generate oxygen.^[^ ^19^ ^]^ Compared to the first two oxygen supply methods, algae has distinct advantages, including high biocompatibility and continuous oxygen production.^[^ ^20^, ^21^ ^]^

Algae, a microorganism discovered in freshwater and seawater, has recently attracted public attention.^[^ ^22^ ^]^ In contrast, certain algae causing environmental issues have been neglected in biomedicine. As prokaryotic photosynthetic microorganisms, cyanobacteria possess chlorophyll and undergo photosynthesis, becoming one of the earliest oxygen producers on Earth.^[^ ^21^, ^23^ ^]^ Their efficient oxygen generation is of particular interest. Therefore, applying photosynthetic cyanobacteria can alleviate tumor hypoxia, improving PDT through photosynthesis.^[^ ^24^, ^25^, ^26^ ^]^ Moreover, cyanobacteria and many of their components can modulate the immune system.^[^ ^27^, ^28^ ^]^ Therefore, compared to other cyanobacteria, Synechococcus sp. exhibits advantages such as high photosynthetic efficiency, rapid growth, strong environmental adaptability, and metabolic diversity, which may make it naturally suitable for PDT usage and immune response activation, enabling the next‐generation microbial tumor nanomedicine development.^[^ ^29^, ^30^ ^]^

**Scheme 1:**
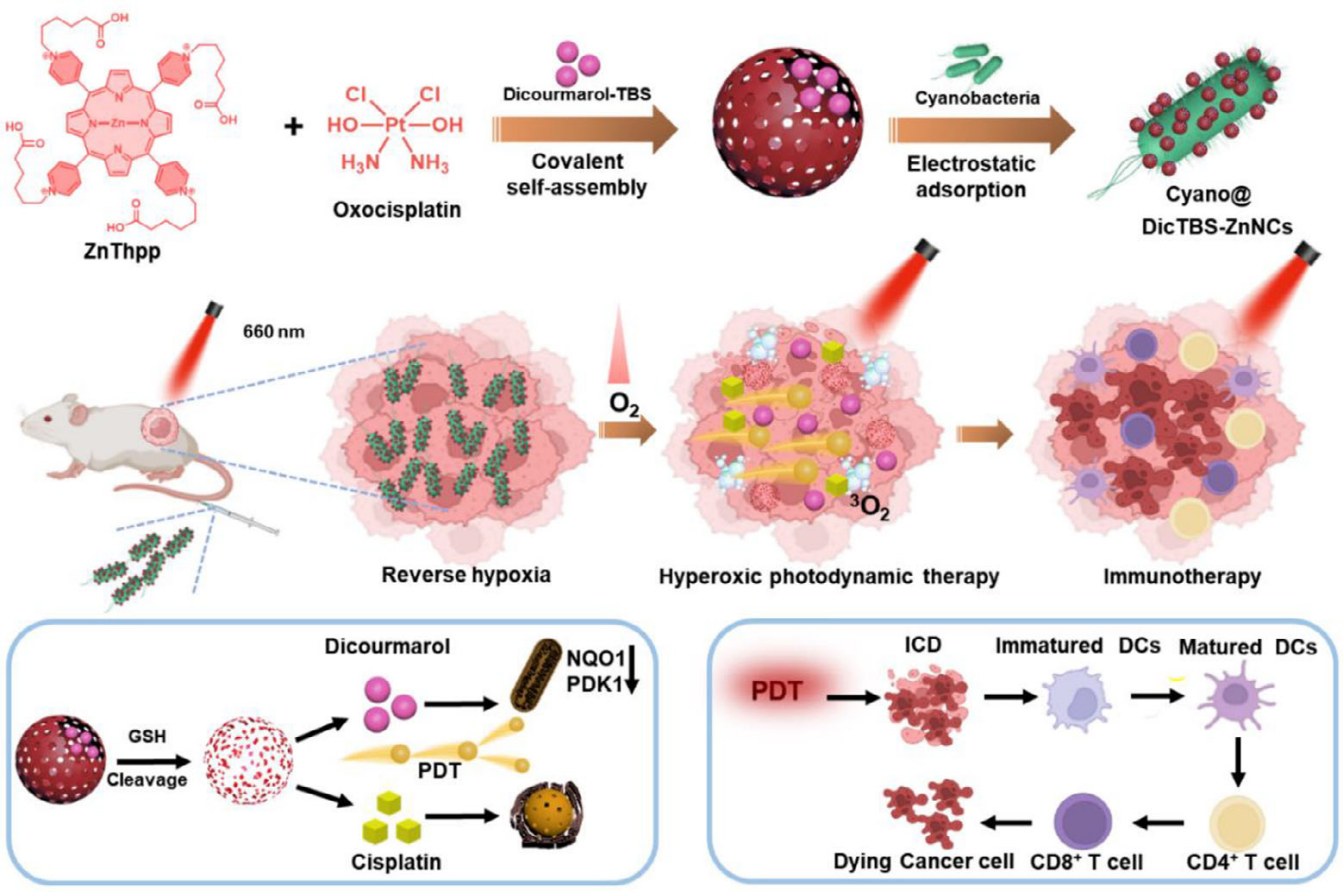
A schematic illustration of the cyanobacteria‐based biohybrid platform for photodynamic cancer therapy enhanced PDT. The platform continuously outputs O_2_ (to alleviate tumor hypoxia) and ^1^O_2_ (for tumor treatment), achieving improved photodynamic immunotherapy.

Recently, polymer nanocapsules (NCs) with hollow interiors and ultra‐thin shells, formed using covalent self‐assembly of building blocks and connecting bonds, have garnered significant attention because of their potential applications.^[^ ^31^, ^32^ ^]^ Since Kim et al.^[^ ^33^ ^]^ invented covalent self‐assembled polymer nanocapsules (NCs), this technology has depicted significant potential in diverse applications, including drug delivery,^[^ ^34^, ^35^ ^]^ bioimaging,^[^ ^36^ ^]^ and catalysis.^[^ ^37^ ^]^ Particularly, the surface of NCs formed by covalent assembly possesses great potential for modification and can be derived for targeting, imaging, catalysis, and other functions. This study developed a novel synergistic functional living biotherapeutics (Cyano@DicTBS‐ZnNCs) using electrostatic adsorption of prodrug nanocapsules on cyanobacteria, leading to multi‐model synergistic therapy. The extensibility and modifiability of Cyano were preliminarily confirmed. First, reduction‐responsive covalent nanocapsules containing the platinum prodrug were constructed according to the porphyrin derivative (ZnThpp) and oxidized cisplatin (Oxocisplatin). Furthermore, the dicumarol derivative (DicTBS), a dual inhibitor of NAD(P)H:quinone oxidoreductase 1 (NQO1) and pyruvate dehydrogenase kinase 1 (PDK1), was encapsulated inside the nanocapsules to improve antitumor efficiency.^[^ ^38^, ^39^, ^40^, ^41^ ^]^ NQO1 protects cells from the harmful effects of quinone redox cycling and their capacity to consume glutathione. This is considered a “signature cell protective enzyme.” PDK1 is an important mediator of cellular respiration. Inside the tumor cells, the nanocapsules collapsed in the presence of over‐expressed GSH to chemotherapeutic cisplatin and porphyrin derivative, releasing DicTBS cargoes. Besides, under the low pH conditions of TME, the inactive DicTBS can be hydrolyzed into dicumarol to inhibit NQO1 and PDK1 against cancer cells. Moreover, cyanobacteria and highly integrated covalent capsules have new functions in different studies. Cyano@DicTBS‐ZnNCs enhanced antitumor properties for PDT and possessed immunomodulatory effects. Immunogenic cell death (ICD) is a type of tumor cell death that initiates immune responses against tumors using various factors. PDT can induce ICD and modulate TME, promoting an immune‐supportive environment and enhancing the antitumor immune response. (**Scheme** **1**).

## 2 Results and Discussion

### 2.1 The Preparation and Characterization of Cyano@DicTBS‐ZnNCs

**Figure 1:**
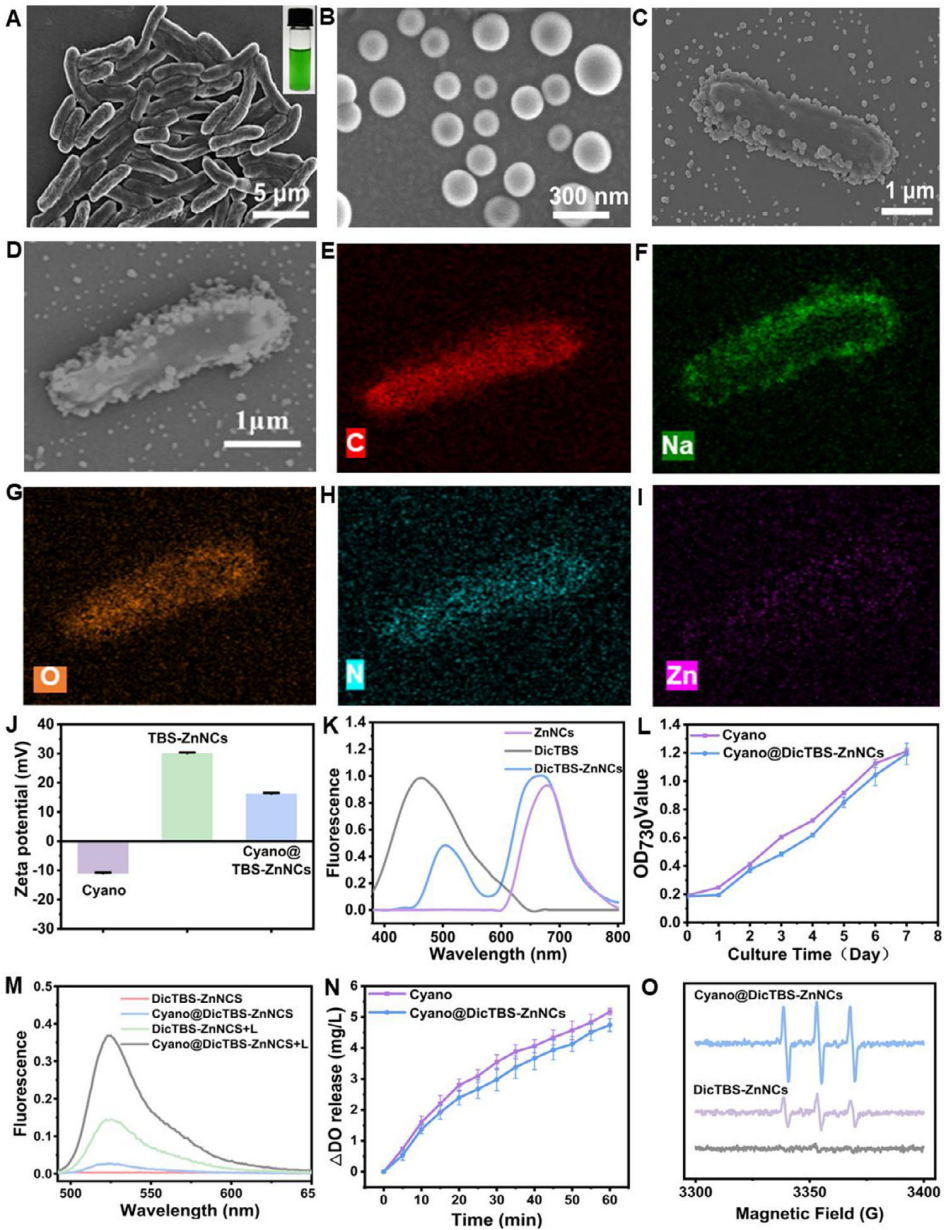
The preparation and characterization of Cyano@DicTBS‐ZnNCs. A) SEM and optical images of Cyanobacteria. B) SEM image of DicTBS‐ZnNCs. C) SEM image of Cyano@DicTBS‐ZnNCs. The scale bar is 1 µm. D) SEM and corresponding EDS of Cyano@DicTBS‐ZnNCs: E (C), F (Na), G (O), H (N) and I (Zn). J) The zeta potential of Cyano, DicTBS‐ZnNCs, and Cyano@DicTBS‐ZnNCs (*n* = 3). K) The fluorescence spectra of ZnNCs, DicTBS, and CyanoTBS‐ZnNCs. L) The growth curve of Cyano and Cyano@DicTBS‐ZnNCs (*n* = 3). (M) Fluorescence spectrum at 524 nm of DCFH treated of DicTBS‐ZnNCs, Cyano@DicTBS‐ZnNCs, DicTBS‐ZnNCs+L, and Cyano@DicTBS‐ZnNCs+L under 660 nm irradiation (300 mW cm^−2^, 10 min). N) The oxygen production curves of Cyano and Cyano@DicTBS‐ZnNCs treated using 660 nm laser irradiation (*n* = 3). O) ESR spectra depicting the ^1^O_2_ produced by Cyano@DicTBS‐ZnNCs and DicTBS‐ZnNCs under 660 nm irradiation with the DMPO probe.

The cyanobacterial (Cyano) was transferred into BG‐11 medium, cultivating at 28 °C under phototrophic conditions for three days in an illuminated incubator. Subsequently, the cyanobacteria are harvested using centrifugation, washed with Phosphate‐Buffered Saline (PBS), and resuspended in PBS to retrieve a cyanobacterial suspension. The morphological characteristics of the Cyano were examined using Scanning Electron Microscopy (SEM) and Transmission Electron Microscopy (TEM). **Figures** **1**A and S1 (Supporting Information) demonstrate the canonical bacilliform morphology of the Cyano. Chlorophyll A imparts a green hue to the Cyano suspension (Figure 1A). Cyano contains chlorophyll A, which exhibits distinct absorption peaks in the blue‐violet and red light regions (Figure S2, Supporting Information). We successfully synthesized ZnThpp and Oxocisplatin using synthetic routes (Figures S3 and S4, Supporting Information), which served as the building blocks and linking units to form covalent capsules. Furthermore, we formed reduction‐responsive covalent nanocapsules, denoted as ZnNCs in Figure S5 (Supporting Information), by cross‐linking these two components. During synthesis, incorporating DicTBS yields nanocapsules encapsulated with DicTBS, referred to as DicTBS‐ZnNCs (Figure S6, Supporting Information). Figure 1B depicts that DicTBS‐ZnNCs are uniformly sized and spherical in shape. TEM images complement the nanocapsule characteristics, such as their hollow internal structure and ultra‐thin wall thickness (Figure S7, Supporting Information). After encapsulating the Dic‐TBS, the images demonstrate no significant changes in the shape and size of the nanocapsules (Figure S8, Supporting Information). The size of the nanocapsules is ≈180 nm. The fluorescence spectra of ZnNCs, DicTBS, and DicTBS‐ZnNCs were measured (Figure 1K). The fluorescence spectrum of DicTBS‐ZnNCs possesses peaks like those of ZnNCs. Additionally, it exhibits characteristic peaks of DicTBS, depicting its successful loading. Cyano@DicTBS‐ZnNCs exhibited an intermediate zeta potential between cyanobacteria and DicTBS‐ZnNCs. (Figure 1J). DicTBS‐ZnNC combined on Cyano was observed by SEM (Figure 1C) and TEM imaging (Figure S9, Supporting Information). Further, energy‐dispersive X‐ray spectroscopy (EDS) elemental mappings of Cyano@DicTBS‐ZnNCs reveal the uniformly distributed C, Na, O, N, and Zn elements throughout the entire architectures (Figure 1D–l). Inductively coupled plasma mass spectrometry (ICP‐MS) indicates that the Zn loading in the samples increases gradually with the concentration of DicTBS‐ZnNCs (Figure S10, Supporting Information). Cyano@DicTBS‐ZnNCs displayed a significant adhesion of DicTBS‐ZnNCs on its surface. These results imply the successful integration of DicTBS‐ZnNCs with Cyano. Later, we investigated whether modifying DicTBS‐ZnNCs could affect the growth, viability, and function of constructed Cyano. Interestingly, Cyano and Cyano@DicTBS‐ZnNCs exhibited comparable growth curves in the BG‐11 medium. This was indicated by the absorbance values at OD730 (Figure 1L). The oxygen production of DicTBS‐ZnNCs and Cyano@DicTBS‐ZnNCs using a 660‐nanometer laser was analyzed and compared. The results indicated that the loading of ZnNCs did not affect oxygen generation. Moreover, oxygen production is positively associated with Cyano quantity. The dissolved oxygen concentration in water is 5–6 mg L^−1^ at room temperature, suggesting that Cyano has enhanced the dissolved oxygen content in water by ≈60% at high concentrations. Therefore, the active hybrid system can perform photoautotrophic and oxygenic photosynthesis for oxygen production, ensuring its function as a new oxygen production system. (Figure 1N). ZnNCs, synthesized using porphyrin derivatives, exhibit photodynamic therapy (PDT) properties. We evaluated the reactive oxygen species (ROS) generation capability of ZnNCs under light exposure (660 nm, 10 min, 300 mW cm^−2^) using DCFH as a fluorescent probe for ROS. The Cyano@ZnNCs + Light group depicted a higher fluorescence intensity at 524 nm, indicating ROS production (Figure 1M). The fluorescence curves under varying light intensities and experimental durations in Figure S11 (Supporting Information) confirmed an increase in fluorescence. This suggested a correlation with enhanced ROS generation. ESR spectroscopy confirmed ROS generation (Figure 1O), particularly within Cyano@ZnNCs, where the signal peak was significantly enhanced. This enhancement is because Cyano supplies oxygen via photosynthesis. Then, it is converted into singlet oxygen using the hybrid system under light, depicting a synergistic effect. This oxygen‐producing PDT platform has significant potential to treat hypoxic tumors.

### 2.2 In Vitro Synergistic Tumor Therapy of Cyano@DicTBS‐ZnNCs

**Figure 2:**
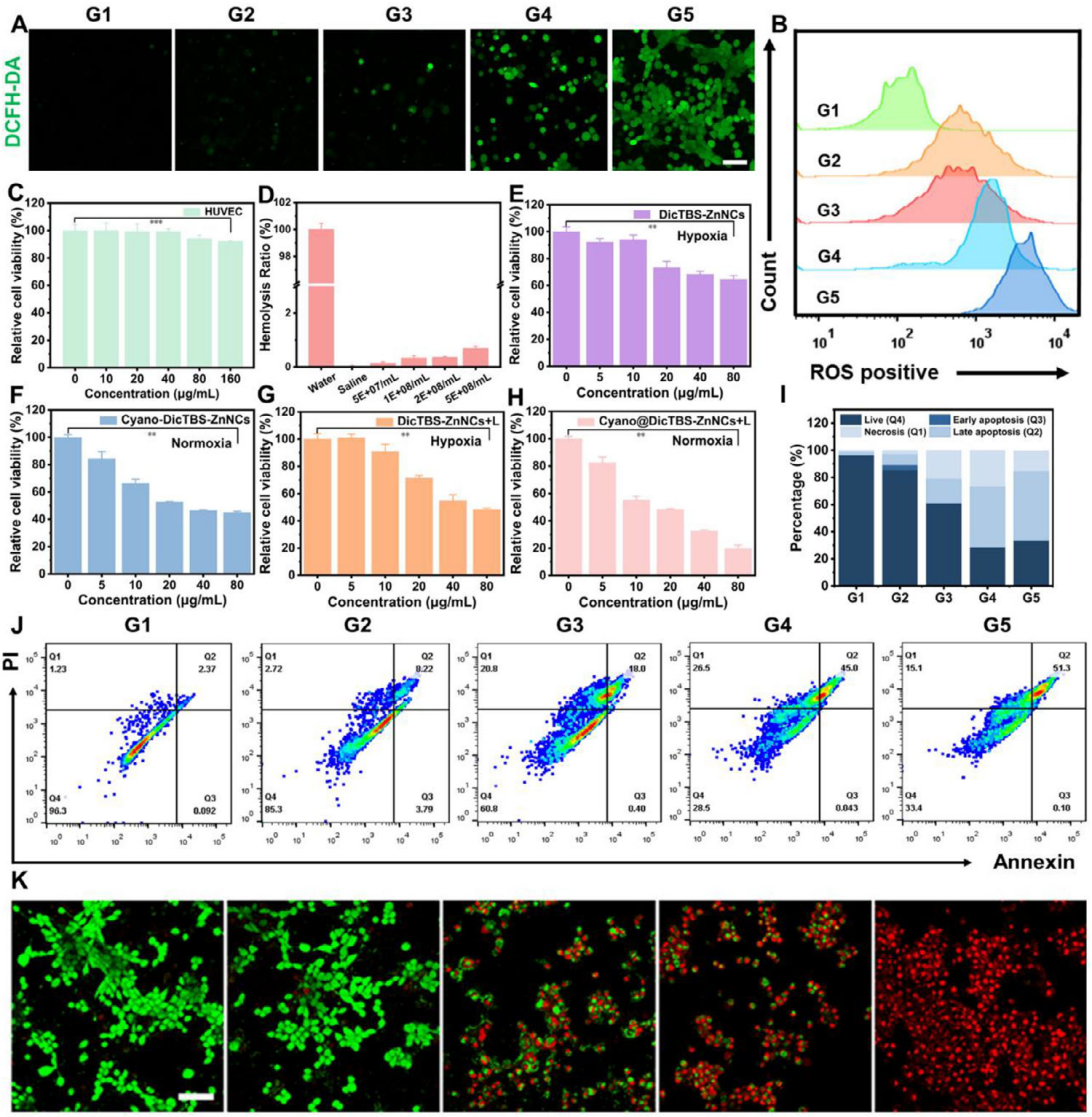
In vitro cellular experiments. A) CLSM images and B) flow cytometry analysis of 4T1 cells stained using DCFH‐DA to reflect the intracellular ROS level after treatment with PBS + L (G1), ZnNCs (G2), DicTBSZnNCs (G3), DicTBSZnNCs + L(G4), and Cyano@DicTBSZnNCs + L(G5). C) The relative cell viabilities of HUVEC cells after incubating with different Cyano@DicTBSZnNC concentrations (*n* = 3). D) Hemolysis rate of mouse red blood cells after incubation with Cyano@DicTBS‐ZnNCs under various concentrations. The relative cell viabilities of 4T1 cells after incubating with different concentrations of E) DicTBS‐ZnNCs, F) Cyano@DicTBS‐ZnNCs, G) DicTBS‐ZnNCs + L, and H) Cyano@DicTBS‐ZnNCs + L.(I) The quantitative analysis of live cells and apoptosis degree of the results in (J). J) Flow cytometry of 4T1 cells using Annexin V‐FITC and PI staining and K) live/dead staining images of 4T1 cells with calcein‐AM (green) and PI (red) test kits treated using different formulations. Scale bar = 100 µm, and the data represent mean ± SD (*n* = 3). ^*^ *p* < 0.05, ^**^ *p* < 0.01, ^***^ *p* < 0.001.

We conducted experiments to evaluate the therapeutic efficiency of Cyano@DicTBS‐ZnNCs in vitro after successfully synthesizing Cyano@DicTBS‐ZnNC biohybrids. This study assessed the anticancer effects of Cyano@DicTBS‐ZnNCs using a mouse mammary carcinoma cell line, 4T1, as the representative model. Five distinct groups could be established for in vitro examination of the synergistic therapeutic impact of the Cyano@DicTBS‐ZnNCs to classify the effects: Group 1 (G1) involved using PBS with light (L); Group 2 (G2) incorporated PBS with ZnNCs; Group 3 (G3) featured DicTBS‐ZnNCs; Group 4 (G4) used the DicTBS‐ZnNCs with light (L); and Group 5 (G5) applied the Cyano@DicTBS‐ZnNCs using light (L) exposure. This structured approach aimed to dissect the contributions of each component involving the overall tumor treatment strategy. The cellular generation of ^1^O_2_ using Cyano@DicTBS‐ZnNCs upon laser irradiation (660 nm, 300 mW cm^−2^) could be quantitatively detected with a fluorescence probe 2ʹ,7ʹ‐dichlorofluorescein diacetate (DCFH‐DA). This could convert into 2ʹ,7ʹ‐dichlorofluorescein (DCF) having a strong green fluorescence in the presence of ^1^O_2_ species. The confocal images (**Figure** **2**A) indicated that no fluorescence signal from DCF could be observed in G1. Weak fluorescent signals from DCF were observed in G2 and G3 without light irradiation. However, fluorescence signals were detected in cells treated using G4 with light irradiation. In contrast, a significant increase in fluorescence signal in G5, with cells treated using Cyano, depicted that the therapeutic efficiency of photodynamic therapy could be highly dependent on the O_2_ level. Cyano@DicTBS‐ZnNCs based on PDT demonstrate the potential for excellent therapeutic efficiency in hypoxic TME. Meanwhile, quantitative flow cytometric analysis of cellular fluorescence exhibited consistent results (Figure 2B).

We conducted experiments to evaluate the therapeutic efficacy of Cyano@DicTBS‐ZnNCs‐based photodynamic therapy in vitro using the successfully synthesized Cyano@DicTBS‐ZnNCs biohybrid. Due to the highly hypoxic nature of the actual tumor microenvironment, all the cell culture experiments were performed in a hypoxic incubator with an oxygen concentration of 2% to decipher their PDT efficiency. First, the standard methyl thiazolyl tetrazolium (MTT) assay helped measure the cytotoxicity of Cyano@DicTBS‐ZnNCs to human umbilical vein endothelial cells (HUVEC). Figure 2C revealed negligible cytotoxicity in the tested concentration range when cells were co‐cultured using Cyano@DicTBS‐ZnNCs, depicting good biocompatibility. As depicted in Figure 2D, the hemolysis rate of Cyano@DicTBS‐ZnNCs remained below 1% even at a relatively high concentration (1 mg mL^−1^), suggesting that Cyano@DicTBS‐ZnNCs does not adversely affect the blood cells during circulation. Subsequently, the PDT efficiency of DicTBS‐ZnNCs and Cyano@DicTBS‐ZnNCs was investigated without light irradiation. Figure 2E demonstrates that a DicTBS‐ZnNCs concentration of 80 µg mL^−1^ helped indicate significant cytotoxicity. As illustrated in Figure 2F, the cell survival rate significantly decreased by adding Cyano@DicTBS‐ZnNCs. Therefore, the excellent oxygen‐producing capability of Cyano improves PDT efficiency. Considering the superior PDT effects of porphyrins, we increased light irradiation. The PDT efficiency of DicTBS‐ZnNCs and Cyano@DicTBS‐ZnNCs was significantly enhanced under hypoxic conditions (Figure 2G,H).

Flow cytometry staining using Annexin V‐FITC and PI revealed that the apoptosis rate within the group treated with laser‐irradiated Cyano@DicTBS‐ZnNCs hybrid (G5) was 51.30%. In contrast, the rates for groups G1, G2, G3, and G4 were 2.37%, 8.22%, 18.00%, and 45.00%, respectively (Figure 2I,J). Thus, live/dead co‐staining analysis using calcein‐AM/propidium iodide (PI) (Figure 2K) showed that compared to other groups, group G5 depicted significant cell death in the live/dead co‐staining assay. This was attributed to the superior photodynamic effect of the hybrid. To investigate the drug delivery efficacy of the biohybrids, we employed laser CLSM to examine their cellular internalization process. ZnNCs contain porphyrin groups, which confer red fluorescence properties. Meanwhile, Hoechst 33 342 (a commercially available blue fluorescent dye) can penetrate the cell membrane and bind to DNA. As shown in Figure S12 (Supporting Information), after treatment, ZnNCs exhibited strong red fluorescence in the cytoplasm, indicating that ZnNCs were successfully internalized by live cells and localized within the cytoplasm.

### 2.3 In Vivo Synergistic Antitumor Efficiency

**Figure 3:**
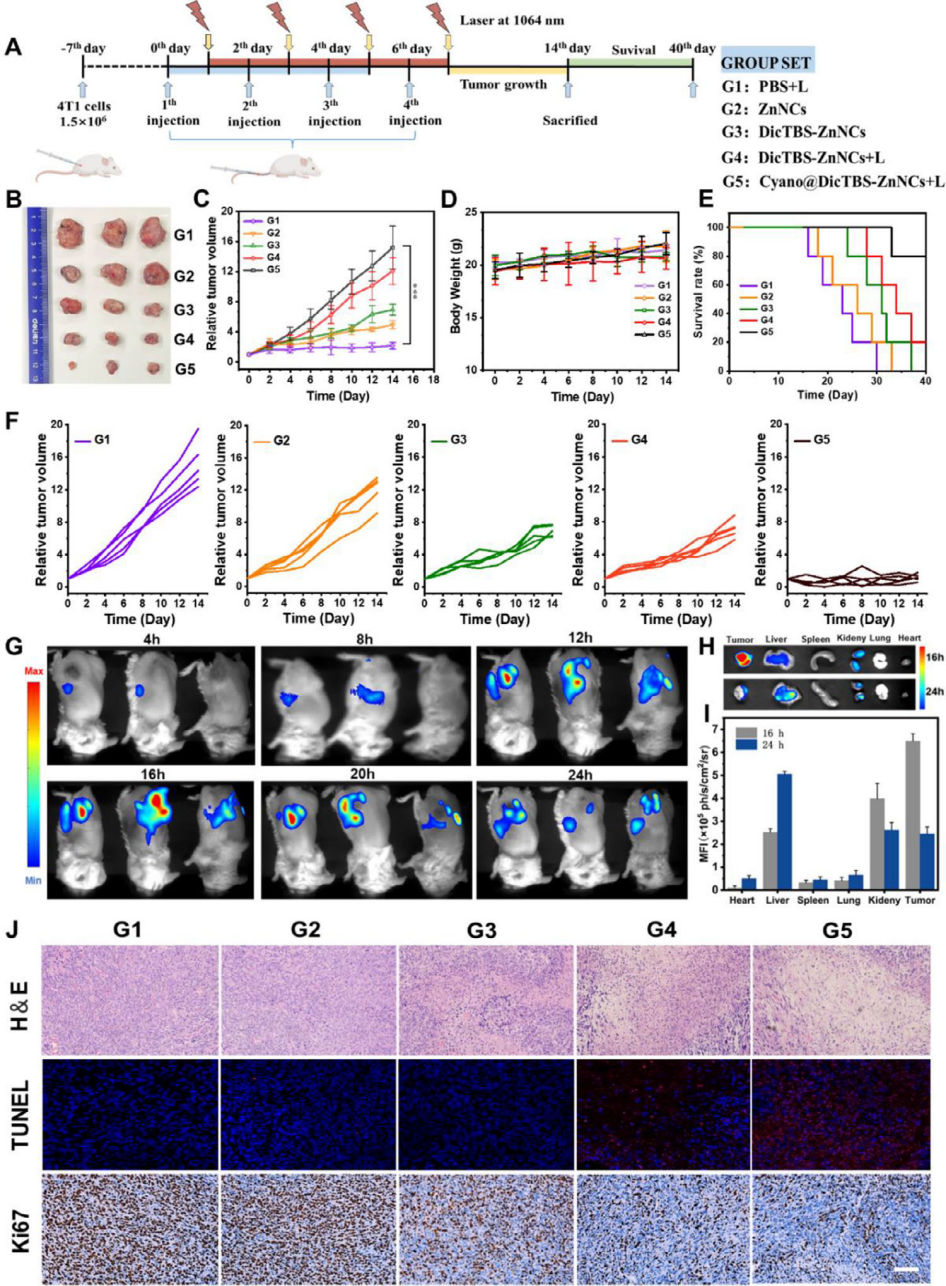
In vivo synergistic antitumor therapy A) The schematic illustration of Cyano@DicTBS‐ZnNCs therapy based on a 4T1‐bearing mouse model. The mice were administered using PBS + L (G1), ZnNCs (G2), DicTBS‐ZnNCs (G3), DicTBS‐ZnNCs +L (G4), and Cyano@DicTBS‐ZnNCs + L (G5), on 0th, 2nd, and 4th day. Moreover, tumor volume and survival rate were recorded on the 14th and 40th day. B) The photograph of isolated 4T1 tumors of mice on the 14th day. C) Tumor growth curves. D) Body weights. E) Kaplan‐Meier survival plots of mice. F) Tumor growth curves of each mouse after different formulations (*n* = 3). The irradiation density was 300 mW cm^−2^ at 660 nm with an irradiation time of 10 min. G) In vivo bioluminescence images of the mice at different time points to track the Cyano@DicTBS‐ZnNCs. H) Ex vivo fluorescence imaging of tissues 16th and 24th hours postinjection and I) corresponding tissue MFI values. J) H&E, TUNEL, and Ki67 staining of tumor tissues obtained from the mice administrated with different formulations. ^*^ *p* < 0.05, ^**^ *p* < 0.01, ^***^ *p* < 0.001. The scale bar is 100 µm, and the data are represented as mean ± S.D. (*n* = 3).

Encouraged by the outstanding in vitro results, the in vivo synergistic anticancer effects of Cyano@DicTBS‐ZnNCs were investigated in the tumor‐bearing mice model. **Figure** **3**A schematically illustrated that the 4T1 tumor‐bearing mice model was created through direct subcutaneous implantation of ≈1.5 × 10^6^ 4T1 tumor cells per BALB/c nude mice on the seventh day. When the tumor volume reached 70 mm^3^, the 4T1 tumor‐bearing mice were randomly divided into five groups while treated with PBS + L (G1), DicTBS‐ZnNCs (G2), DicTBS‐ZnNCs + L (G3), Cyano@DicTBS‐ZnNCs (G4), and Cyano@DicTBS‐ZnNCs + L (G5) using in situ injection on the 0th, 2nd, 4th, and 6th day (10^7^ CFU mL^−1^), respectively. The tumors were exposed to 660 nm laser irradiation at 16 h for PDT after i.v. injection. After 14 days of treatment, the visual differences for the tumor structures could be seen (Figure 3B). The tumor treated with PBS (Group G1) exhibited a 15.1‐fold elevation in tumor volume despite light irradiation, suggesting the rapid growth and malignancy of tumor cells. In contrast, Groups G2 and G3 demonstrated antitumor effects, with tumor volumes rising by 12.07‐fold and 6.92‐fold, respectively. This indicates the PDT efficacy of ZnNCs and the potent antitumor toxicity of DicTBS. When Group G4 was treated using laser irradiation, this increase was significantly reduced to 4.9‐fold. Adding Cyano in Group G5 enhanced the PDT effect, with the tumor volume increasing only 2.16‐fold (Figure 3C,F). Combined with the strong antitumor toxicity of DicTBS, the enhanced oxygen generation using cyanobacteria, which boosts the PDT effect of ZnNCs, significantly improves the outstanding therapeutic outcomes in cancer treatment. Dynamic monitoring of the mice's body weight suggested no significant weight loss during the 14‐day treatment period, with minimal side effects of the materials on healthy organs (Figure 3D). Consistent with the tumor growth inhibition results, the Kaplan‐Meier survival curve analyses depicted that the survival time of mice in Group G5 was significantly extended compared to other groups (Figure 3E). After a 40‐day evaluation, Group G6 effectively prolonged the survival of mice, achieving the highest survival rate of 80%. In contrast, other groups (from G1 to G5) had complete mortality (0% survival rate).

Cyano@DicTBS‐ZnNCs labeled with Cy5.5 were used to elucidate the temporal biodistribution. The experiment demonstrated the fluorescence intensity of the tumor over time, with the fluorescence signal in the tumor reaching its peak 16 h after intravenous injection of Cyano@DicTBS‐ZnNCs. This suggests maximum cyanobacteria accumulation and helps determine the optimal timing for photodynamic therapy (Figure 3G). We collected mice tumors and major organs for ex vivo fluorescence imaging at 16th and 24th hours postintravenous injection (Figure 3H). The results indicated that the fluorescence intensity in tumors treated at the 16th hour was significantly higher than that in tumors treated at the 24th hour. Notably, the fluorescence in the liver and kidneys was higher compared to other organs (Figure 3I), which may be attributed to the metabolic processing of the drugs by these organs. Furthermore, hematoxylin and eosin (H&E) staining demonstrated that G5 demonstrated extensive tissue necrosis and nuclear pyknosis across a large tumor area after 14 days of treatment. In contrast, the control groups showed little difference (Figure 3J). Moreover, terminal deoxynucleotidyl transferase‐mediated dUTP nick end‐labeling (TUNEL) and Ki67‐positive immunohistochemical staining detected severe apoptotic damage (purple spots) and the lowest proliferation (brown spots) within the tumor tissues of Group G5, respectively. This demonstrated the significant anticancer capability of the nanomotors in vivo. Even with laser irradiation after 14 days of treatment, the Cyano@DicTBS‐ZnNCs biohybrid showed negligible effects on the main organs (heart, liver, spleen, lung, and kidney) of the mice (Figure S27, Supporting Information).

**Figure 4:**
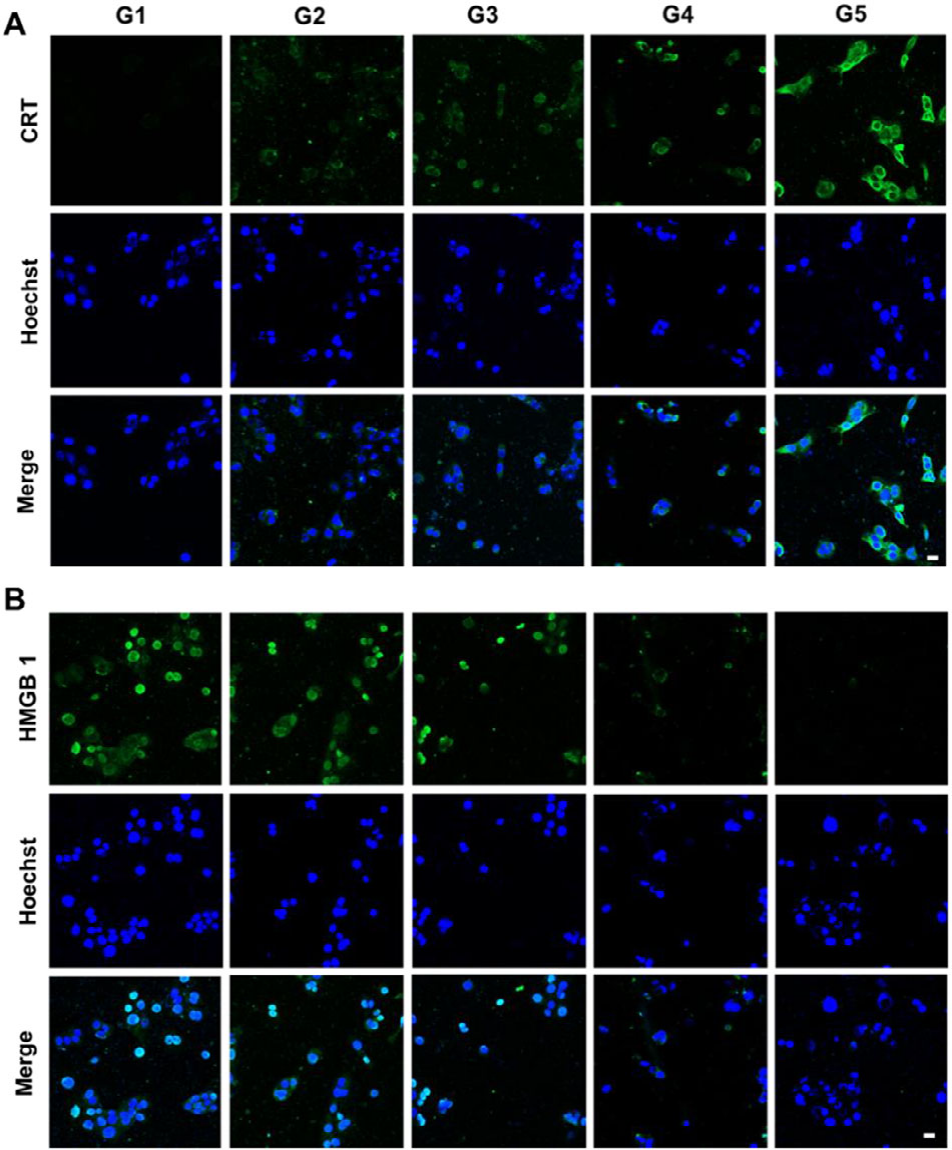
CLSM images of cells showing A) CRT exposure and B) HMGB1 release after treatment with the corresponding drugs for 20 h (scale bar: 20 µm).

ICD represents a programmed cell death pathway characterized by three key hallmarks: 1) adenosine triphosphate (ATP) release, 2) surface translocation of calreticulin (ecto‐CRT), and 3) high mobility group box 1 (HMGB1) expression. These danger signals promote dendritic cell maturation and antitumor T cell activation, establishing potent antitumor immunity. Notably, photodynamic therapy (PDT) particularly efficiently induces damage‐associated molecular pattern (DAMP) generation through oxidative stress mechanisms. As shown in **Figure** **4**A,B, compared to those in the control groups, cells treated with G5 demonstrate these characteristic ICD features: significantly increased ecto‐CRT expression, elevated extracellular HMGB1 release, and markedly reduced intracellular ATP stores (Figure S28, Supporting Information). This triad of biomarkers confirms successful ICD induction by our treatment protocol.

### 2.4 Tumour Immune Responses After PDT Using Cyano@DicTBS‐ZnNCs

**Figure 5:**
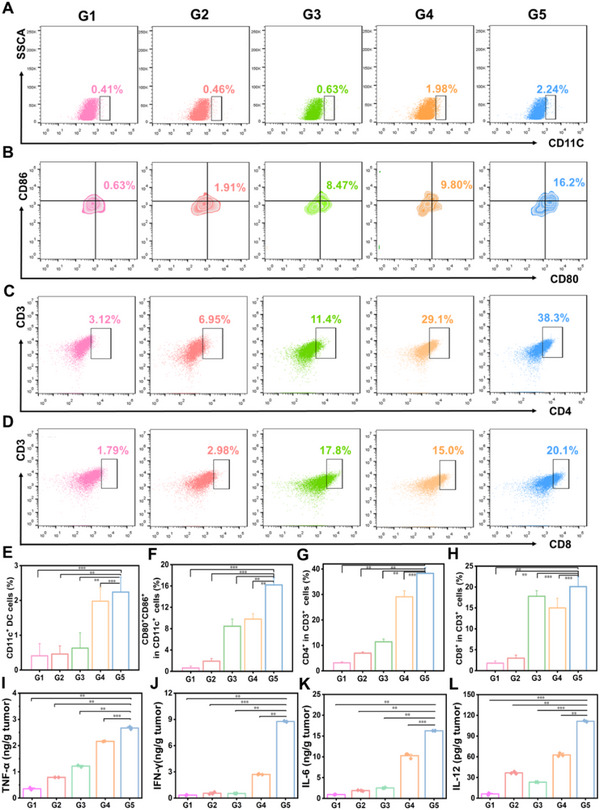
Tumour immune responses after PDT with Cyano@DicTBS‐ZnNCs. A,B) Flow cytometry analysis of matured DCs (CD80^+^CD86^+^ on gated on CD11c^+^ DCs) across different groups post‐therapy. C) Flow cytometry analysis of CD4^+^ T cells and D) CD8^+^ T cells (gated on CD3^+^ T cells) across different groups post‐therapy. The quantitative analysis of E,F) DCs maturation, G) CD4^+^ T cells, and (H) CD8^+^ T cells with matured secretion in different groups post‐therapy. The levels of I) TNF‐α, J) IFN‐γ, K) IL‐6, and L) IL‐12 in tumors with different treatments as indicated (*n* = 3). ^*^ *p* < 0.05, ^**^ *p* < 0.01, ^***^ *p* < 0.001.

The anti‐tumor immune response in 4T1 tumor‐bearing mice was examined to uncover the underlying mechanism of PDT mediated by Cyano@DicTBS‐ZnNCs in vivo. Immune cells were collected and analyzed from the tumor tissues of various groups. Based on the in vitro results of DC maturation, DC maturation stimulated by different treatments was investigated. CD11c is a surface marker of dendritic cells (DCs) and can help identify them. CD80 and CD86, as co‐stimulatory molecules, can evaluate DC maturation. The flow cytometry analysis (**Figure** **5**A,E) showed that the relative number of mature DCs (CD11c^+^) was significantly elevated in the G5 group postlaser irradiation. Additionally, the proportion of mature DCs (CD11c^+^/CD80^+^CD86^+^) in the G5 group rose to 16.2%, significantly higher than in other groups (Figure 5B,F). These results were consistent with the in vitro experimental results, validating that ZnNCs can confer immunomodulatory properties to Cyano and activate a strong immune response in vivo. Moreover, flow cytometry analysis established the presence of CD4^+^ T‐helper cells and CD8^+^ cytotoxic T lymphocytes in the immunological milieu of the tumor, eliminating malignant cells. A notable escalation in the frequency of both CD4^+^ T‐helper cells and CD8^+^ IFN‐γ positive cytotoxic T lymphocytes (CTLs) could be observed in tumor tissues post‐treatment using the Cyano@DicTBS‐ZnNCs regimen coupled with photoirradiation (Figure 5C,D,G,H). This is indicative of a robustly triggered immune response against the tumor. These findings align with the therapeutic strategies to promote dendritic cell maturation, pivotal for initiating an effective immune reaction. Based on these findings, it is evident that Cyano@DicTBS‐ZnNCs displayed noticeable immunomodulatory capabilities, significantly contributing to improved therapeutic outcomes. In addition to different immune cells in the tumor, the levels of diverse pro‐inflammatory cytokines including TNF‐α, IFN‐γ, IL‐6, and IL‐12 were also significantly increased in Cyano@DicTBS‐ZnNCs PDT treatment (Figure 5I–L). All the above results strongly evidenced that such bacterial biohybrids plus Light irradiation could collectively elicit a robust antitumor immune response.

### 2.5 Transcriptomic Analysis

**Figure 6:**
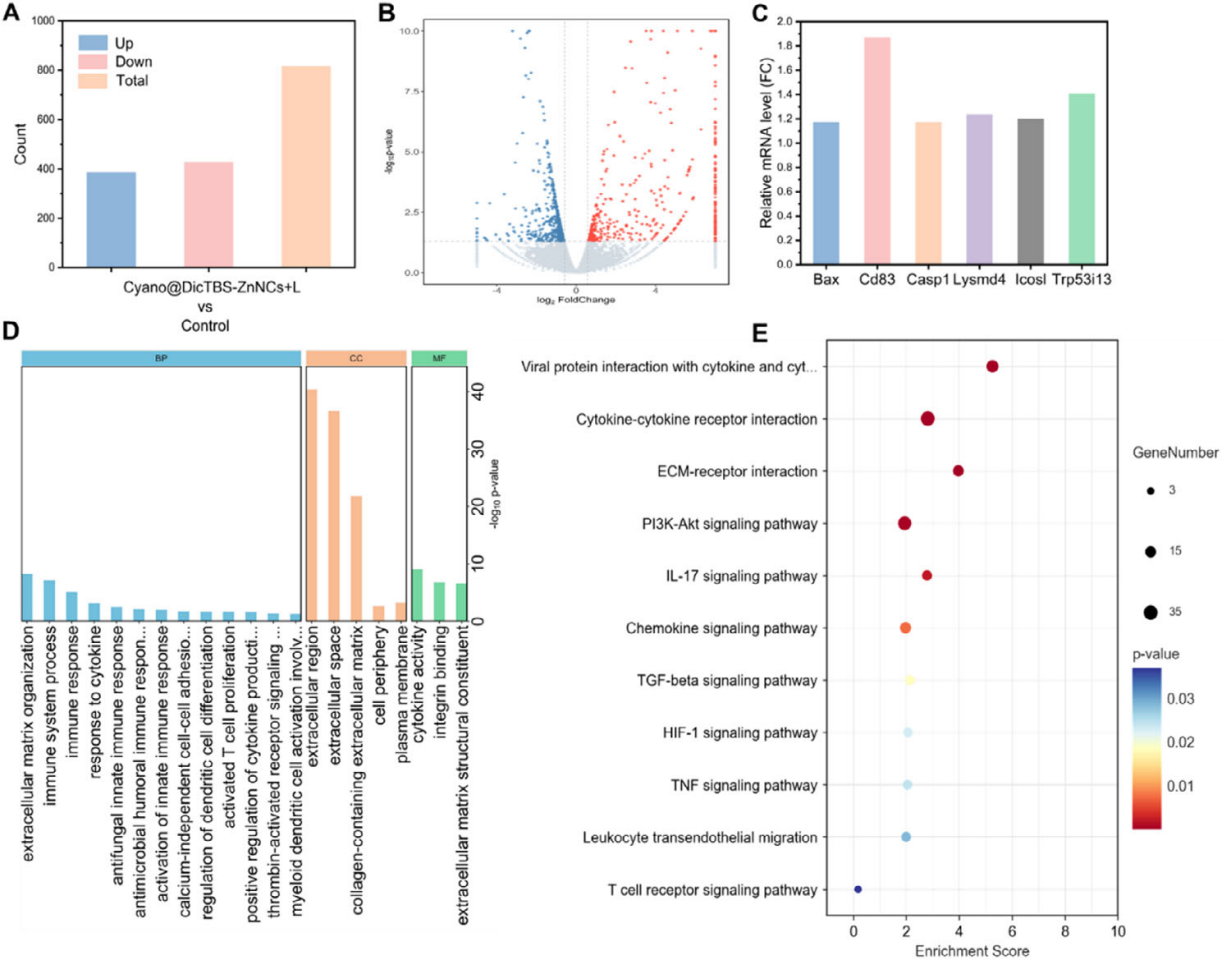
Transcriptomic analysis of 4T1 tumor tissues after PBS and Cyano@DicTBS‐ZnNCs PDT treatment. A) The differential expressed genes (DEGs) in (Cyano@DicTBS‐ZnNCs) group and the control group. B) Volcano plot of all the DEGs between the (Cyano@DicTBS‐ZnNCs) group and the control group. C) Relative mRNA expression levels of selected genes following photodynamic therapy (PDT). The fold change (FC) between the (Cyano@DicTBS‐ZnNCs) group and the control group is shown. D) Histogram presenting the GO enrichment analysis results of some selected DEGs between the treated (Cyano@DicTBS‐ZnNCs) group and the control group. E) Dot plot illustrating the KEGG enrichment analysis results of some selected DEGs between the treated (Cyano@DicTBS‐ZnNCs) group and the control group.

To elucidate the underlying mechanisms of the immunostimulatory effects of the biohybrid photodynamic therapy strategy, we conducted a transcriptomic analysis on 4T1 tumor tissues. Specifically, we designated the tumor tissues from mice bearing 4T1 tumors treated with PBS as the control group, and those treated with Cyano@DicTBS‐ZnNCs as the experimental group. The volcano plot results showed a total of 815 differentially expressed genes of which 387 were upregulated and 428 were downregulated (**Figure** **6**A,B), indicating significant changes in the tumor after PDT treatment facilitated by the biohybrid. Subsequently, we performed GO (Gene Ontology) and KEGG enrichment analyses on the tumor tissues to identify notable changes following PDT treatment. As shown in the GO enrichment analysis results (Figure 6D), the biological processes (BP) of tumor cells after treatment were significantly affected. The composition and organization of the extracellular matrix (ECM) in the cellular component (CC) category were also affected due to the increase in oxygen. Importantly, we found that most of the differentially expressed genes were enriched in signaling pathways related to immune responses, including immune system process, response to cytokines, myeloid dendritic cell activation, dendritic cell differentiation, activated T cell proliferation, immune response regulation, and cytokine activity, indicating that the PDT treatment enhanced by Cyano@DicTBS‐ZnNCs has an immunostimulatory effect. The KEGG enrichment analysis results confirmed the activation of immune response‐related signaling pathways after treatment (such as PI3K‐Akt, IL‐17, TGF‐beta, HIF‐1, TNF, and many other signaling pathways, T cell receptor signaling pathways, and cytokine‐cytokine receptor interactions) (Figure 6E). In addition, we quantitatively characterized the relative mRNA expression levels (fold change, FC) of several key genes, including *Bax*, *Cd83*, *Casp1*, *Lysmd4*, Icosl, and *Trp53i13*, as shown in Figure 6C, to evaluate the differences in gene expression between the G5 and control group. The results revealed that the genes of *Cd83* and *Trp53i13* were the most significantly upregulated, suggesting enhanced activation of immune and apoptosis‐related pathways. The upregulation of Cd83, a marker of dendritic cell maturation, indicates enhanced antigen‐presenting capacity, which may promote the activation and proliferation of T cells. Meanwhile, the increased expression of Trp53i13 is associated with the activation of the p53 signaling pathway. In summary, the transcriptomic analysis showed significant changes in immune cells in the tumor tissue after treatment. The above results indicate that the biohybrid strategy based on cyanobacteria can reprogram the immunosuppressive tumor microenvironment and induce a strong systemic immune response.

## 3 Conclusion

This study developed a novel living biotherapeutic agent, Cyano@DicTBS‐ZnNCs, to improve photodynamic therapy by covalently displaying a photosensitizer to nanocapsule surface while electrostatically adsorbing it onto oxygen‐producing cyanobacteria. Effective PDT was achieved in a mouse tumor model because of the efficient and extensive distribution of the photosensitive ZnNCs carried using the biohybrid at the tumor site and the sustained tumor hypoxia relief. Furthermore, the PDT of this customized Cyano@DicTBS‐ZnNCs‐based biohybrid could stimulate a strong anti‐tumor immune response. Consequently, this Cyano@DicTBS‐ZnNCs‐based PDT depicted significant therapeutic effects while suppressing tumor growth. Future work will focus on optimizing the delivery system and exploring its application in other cancer models.

## 4 Experimental Section

### Chemicals

The experimental reagents and materials used were commercially purchased without further purification. All the solvents, such as N, N‐dimethylformamide (DMF), ether, acetonitrile, propionic acid, methanol, and dichloromethane (DCM), were obtained from Beijing Chemical Factory. N, N‐dimethylformamide (DMF), and acetonitrile underwent anhydrous treatment with calcium hydride before use. All the reagents, such as pyrrole, 4‐pyridinecarboxaldehyde, cisplatin, triethylamine (TEA), dithiolbenzoin (Dicourmarol), tert‐butyldimethylchlorosilane (TBSCl), hydrogen peroxide (H_2_O_2_), 4‐dimethylaminopyridine (DMAP), dicyclohexylcarbodiimide (DCC), and 5, 5‐dimethyl‐1‐pyrroline N‐oxide (DMPO), were procured from Sigma‐Aldrich. Agarose was acquired from Sangon Biotech (Shanghai) Co., Ltd. BG‐11 medium and Synechococcus sp (Poly‐Sphera) cyanobacteria were provided by the Freshwater Algae Culture Collection of the Chinese Academy of Sciences. 3‐[4,5‐dimethylthiazol‐2‐yl]‐2,5‐diphenyltetrazolium bromide (MTT) was obtained from Dalian Meilun Biotechnology Co., Ltd. DMEM high glucose medium, fetal bovine serum (FBS), penicillin‐streptomycin mixture, trypsin digestion solution, cell culture dishes, 96‐well plates, DCFH‐DA reactive oxygen species assay kit, 4% paraformaldehyde fixative solution, Hoechst 33342 staining kit, Annexin V‐FITC/PI cell apoptosis detection kit, and Calcein‐AM/PI live/dead cell staining kit were procured from Shanghai Biotech (Shanghai) Co., Ltd. Milli‐Q water was used in the experiments, which was obtained through double‐distillation with 18.2 MΩ cm resistivity.

### Instrumentation

The Bruker Corporation in Germany manufactured the nuclear magnetic resonance spectrometer (AVANCEIII 500), liquid chromatography‐mass spectrometry system (LC‐MS, Agilent 1290‐microTOD‐Q II), and matrix‐assisted laser desorption/ionization time‐of‐flight mass spectrometer (Autoflex speed TOF/TOF). The scanning electron microscope (JSM6700F), transmission electron microscope (JEM‐2100F), and electron spin resonance spectrometer (JES‐FA 200) were developed by JEOL Ltd. in Japan. The UV–visible (3100 UV–vis) and fluorescence (RF‐5301‐PC) spectrophotometers were manufactured by Shimadzu Corporation in Japan. The laser scanning confocal inverted microscope (LSM 710) was developed by Carl Zeiss in Germany. The flow cytometer (BD Accuri C6, USA) was manufactured by Becton, Dickinson Company in the United States. The Malvern Corporation in the UK developed the nanoparticle size analyzer (ZS 90). The microplate reader was manufactured by Nanjing Detie Laboratory Equipment Co., Ltd. The 660 nm near‐infrared laser (FC660LH‐300 mW‐FC) was developed by Changchun New Industries Optoelectronics Tech Co., Ltd. in China. The light incubator (MGC‐250BP‐2) was manufactured by Shanghai Yiheng Technical Co., Ltd., while the dissolved oxygen meter (DO‐957) was developed by Shanghai INESA Scientific Instrument Co., Ltd.

### The Synthetics of Tetrapyridylporphyrin Tpp

The synthesis pathway of ZnThpp is represented in Figure S3 (Supporting Information). Under a nitrogen atmosphere, 4‐Pyridinecarboxaldehyde (60 mmol, 6.42 g) was dissolved in 100 mL of propionic acid and heated to reflux. Subsequently, pyrrole (60 mmol, 4.02 g) was dissolved in 25 mL of propionic acid. This mixture was added dropwise to the aforementioned solution, with the reaction proceeding for 3 h. After completing the reaction, the solvent was removed under reduced pressure, and 100 mL of methanol helped induce recrystallization. Then, the mixture was filtered, and the resulting solid was vacuum‐dried for 24 h, yielding a purple‐black solid (4.12 g, yield: 44.4%). Figure S13 (Supporting Information) indicates that 1H NMR (400 MHz, Chloroform‐d) δ: 9.13–9.08 (m, 8H), 8.91 (s, 8H), 8.23–8.18 (m, 8H), −2.88 (s, 2H).

### The Synthetics of Thpp

Tpp (1 mmol, 618 mg) was dissolved in 20 mL of dry DMF, and 6‐bromohexanoic acid (20 mmol, 3.9 g) was added and reacted at 120 °C for 24 h under a nitrogen atmosphere. After completing the reaction, the filter was cooled and washed with plenty of DMF and DCM. The resulting solid was vacuum‐dried for 24 h. A purple‐black solid was obtained (720 mg, yield: 51.5%). Figure S14 (Supporting Information) shows that 1H NMR (500 MHz, DMSO‐d6) δ: 12.14 (s, 2H), 9.65 (d, J = 6.2 Hz, 8H), 9.27 (s, 7H), 9.06 (d, J = 5.9 Hz, 8H), 5.01 (t, J = 7.6 Hz, 8H), 2.41 (t, J = 7.2 Hz, 8H), 2.37–2.29 (m, 8H), 1.77 (t, J = 7.5 Hz, 8H), 1.65 (t, J = 6.3 Hz, 8H), −3.05 (d, J = 12.6 Hz, 2H). MALDI‐TOF mass spectrum (Figure S15, Supporting Information) m/z: [M + 3H] +calculated for C64H73O8N8, 1081.55; found, 1081.05.

### The Synthesis of ZnThpp

Thpp (1 mmol, 1.398 g) was dissolved in 30 mL of dry DMF, followed by adding zinc acetate (10 mmol, 1.83 g). The reaction was heated at 120 °C for 48 h. After completing the reaction, the mixture was cooled and filtered. The residue was washed using an appropriate amount of cold DMF to remove the excess zinc acetate, followed by washing with DCM. The resulting solid was vacuum‐dried for 24 h to yield a purple‐black solid (1.15 g, yield: 78.7%). As shown in Figure S16 (Supporting Information), 1H NMR (500 MHz, DMSOd6) δ: 9.40 (d, J = 6.1 Hz, 8H), 9.09 (s, 8H), 8.87 (d, J = 5.3 Hz, 8H), 4.92 (s, 8H), 2.30–2.18 (m, 16H), 1.65 (s, 8H), 1.55 (d, J = 6.4 Hz, 8H). MALDI‐TOF mass spectrum (Figure S17, Supporting Information) m/z: [M + 2H]+ calculated for C44H72O8N8Zn, 1142.46; found, 1142.06.

### The Synthesis of Oxocisplatin

The synthesis pathway of oxidized cisplatin is represented in Figure S18 (Supporting Information). Cisplatin (1 mmol, 300 mg) was mixed with 30 mL of hydrogen peroxide (5%) and stirred at 50 °C for 24 h. Subsequently, part of the solvent was removed under reduced pressure to concentrate the mixture. Then, it was placed at 4 °C for 24 h to precipitate a bright yellow solid. The oxidized cisplatin (289 mg, yield: 86.5%) was obtained after filtration. MALDI‐TOF mass spectrum (Figure S19, Supporting Information) m/z: [M]+ calculated for C45H52O10, 332.96; found, 331–335.

### The Synthesis of TBS‐Protected Dicoumarol (DicTBS)

The synthesis pathway of DicTBS is depicted in Figure S20 (Supporting Information). Dicoumarol (5 mmol, 1.68 g) and TBSCl (tert‐Butyldimethylsilyl chloride) (20 mmol, 3.01 g) were dissolved in 50 mL of dry DCM (dichloromethane). After fully dissolving the reagents, dry TEA (triethylamine, 3 mL, 2.07 g) was added and stirred at room temperature for 12 h. After completion of the reaction, the solvent was removed under reduced pressure at a low temperature. The residue was extracted using saturated NaHCO_3_ solution and ethyl acetate, performing three extractions and retaining the organic phase each time. The organic phase was dried using anhydrous Na_2_SO_4_. After removing the solvent, the crude product was obtained, which was purified using silica gel column chromatography to obtain a white solid, DicTBS (1.47 g, yield: 41.5%). 1H NMR (500 MHz, Chloroform‐d) (Figure S21, Supporting Information) δ: 7.74 (dd, J = 8.2, 1.6 Hz, 1H), 7.47 (ddd, J = 8.6, 7.3, 1.6 Hz, 1H), 7.30–7.24 (m, 2H), 3.78 (s, 1H), 1.15 (s, 9H), 0.34 (s, 6H). MALDI‐TOF mass spectrum (Figure S22, Supporting Information) m/z: [M + Na]+ calculated for C31H40O6NaSi2, 587.23; found, 587.43.

### The Synthetics of ZnNCs

We weighed 10 mg of ZnThpp and 9.56 mg of oxocisplatin, followed by dissolving them in 10 mL of anhydrous acetonitrile. Then, 0.7 mg DMAP, 11.7 mg DCC, and 0.5 mL TEA were added, and the mixture was stirred at room temperature for 24 h. Subsequently, the reaction solution was dialyzed against DMF for 24 h, followed by dialysis against water for 48 h, to obtain an aqueous ZnNC solution.

### The Synthetics of DicTBS‐ZnNCs

We weighed 10 mg ZnThpp, 10 mg DicTBS, and 9.56 mg oxocisplatin, followed by dissolving them in 10 mL anhydrous acetonitrile. Then, 0.7 mg DMAP, 11.7 mg DCC, and 0.5 mL TEA were added, and the mixture was stirred at room temperature for 24 h. Subsequently, the reaction solution was dialyzed against DMF for 24 h. This was followed by dialysis against water for 48 h to obtain an aqueous DicTBS‐ZnNC solution.

### The Culture of Cyanobacteria (Cyano)

The cyanobacteria strain (*Synechococcus sp*.) was cultured into BG‐11 medium and incubated in an illuminated incubator at 28 °C for three days (illumination conditions: 2400 Lux; photoperiod: 12 h light/12 h dark). The culture flasks were gently shaken every morning and evening. For subculturing, the cyanobacteria were transferred into fresh sterile BG‐11 medium at a 1:3 volume ratio (this ratio can be adjusted depending on the growth status of the cyanobacteria). The same cultivation conditions were followed as described above. The culture was continued for 3 days until the OD730 reached ≈1.0. This indicated that the cyanobacteria were in the logarithmic growth phase. Then, the cyanobacteria were washed using PBS (10 mm, pH 7.4). The cyanobacteria were centrifuged at 6000 rpm for 2 min, washed twice, and resuspended in PBS to obtain a cyanobacteria suspension.

### In Vitro Oxygen Production by Cyanobacteria (Cyano)

The cyanobacteria suspension, re‐dispersed in PBS, was placed in a dark environment overnight to deplete the dissolved oxygen. The suspension was diluted to an appropriate concentration the next day, followed by measuring the dissolved oxygen concentration. Red light (300 mW cm^−2^) helped illuminate the suspension for 1 h, and the dissolved oxygen content in the cyanobacteria suspension was measured at the same time intervals with a dissolved oxygen meter.

### The Culture of HUVEC and 4T1 Cell Lines

Human umbilical vein endothelial cells (HUVEC) and mouse breast cancer cells (4T1) were cultured in DMEM high‐glucose medium containing 10% fetal bovine serum at 37 °C inside a humidified incubator with 5% CO_2_. When the cells achieved over 90% confluence, they were passaged. The passaging procedure involved discarding the old medium, washing the culture flask three times using PBS, and then adding an appropriate amount of trypsin solution for digestion. After 2 min of digestion, a medium containing fetal bovine serum was added to stop the digestion, gently resuspending the cells and collecting them into a centrifuge tube. The cells were centrifuged at 1000 rpm for 5 min, resuspended, and one‐third of the cell suspension was transferred into a new culture flask to continue culturing.

### Hemolysis Assay

Around 2 mL of blood was drawn from a healthy mouse and diluted with 2.5 mL of physiological saline. Different concentrations of DicTBS‐ZnNCs solutions were prepared with physiological saline and used with deionized water as negative and positive controls, respectively. Around 120 µL of the diluted fresh blood was added to 1 mL of each test sample of different concentrations and gently shaken at 37 °C for 90 min. After centrifuging at 3000 rpm for 10 min, a UV spectrophotometer helped measure the absorbance of the supernatant at 545 nm. The hemolysis rate is calculated with the following formula:

(1) $$\begin{array}{ccc} & & Hemolysis\;rate(\%)\\ & & \;=[(OD\;sample-OD\;negative)/(OD\;positive-OD\;negative)]\times 100\% \end{array}$$

### Cellular Uptake Experiment of ZnNCs

The 4T1 cells were cultured in a confocal culture dish at a density of 5.0 × 10⁴ cells mL^−1^. After incubating for 12 h in the cell culture incubator, the old medium was removed and replaced with a fresh medium containing Cyano@DicTBS‐ZnNCs for co‐incubation for 1 h. The cells were washed three times using PBS. Then, they were co‐incubated with Hoechst 33 342 staining solutions (5.0 µg mL^−1^) for 20 min to stain the cell nuclei. The cells were washed five times with PBS, and a laser confocal inverted microscope helped capture the images.

### Detection of Intracellular ^1^O_2_ Generation

The 4T1 cells were cultured in a confocal culture dish at a density of 5.0 × 10⁴ cells mL^−1^. After incubating for 12 h in the cell culture incubator, the old medium was removed, and the cells were treated in the following ways: PBS + Light (G1), ZnNCs (G2), DicTBS‐ZnNCs (G3), Cyano@DicTBS‐ZnNCs + Light (G4), Cyano@DicTBS‐ZnNCs + Light (G5). After 2 h, DCFH‐DA was added, and the 4T1 cells were irradiated using a 660 nm laser (300 mW cm⁻^2^) for 10 min. Furthermore, the cells were incubated at 37 °C with 5% CO₂ for 1 h. The cells were washed three times using PBS, and a laser confocal inverted microscope helped capture the images.

### Detection of Cell Apoptosis

The 4T1 cells were cultured in a 6‐well plate at a density of 5.0 × 10^4^ cells mL^−1^. After incubating in a cell culture incubator for 12 h, the old medium was removed, and the cells were treated separately: G1, G2, G3, G4, G5. After 4 h, the 4T1 cells co‐incubated with G4 and G5 were irradiated using a 660 nm laser (300 mW cm^−2^, 10 min) and cultured for 20 h at 37 °C with 2% oxygen in the incubator. Cells were harvested using 0.25% trypsin without EDTA and staining with an Annexin V‐FITC/PI apoptosis detection kit. Following this, cells were resuspended in 1× binding buffer (Invitrogen) at a concentration of 1 × 10⁶ cells per mL. Annexin V‐FITC (5 µL) and propidium iodide (PI) (5 µL) were added to the cell suspension and incubated for 15 min at room temperature in the dark. Finally, cell apoptosis was evaluated using a flow cytometer.

### Animals and Tumor Model

All animal experiments were carried out in the Laboratory Animal Center of Hangzhou Normal University and were approved by the Ethics Committee of Laboratory Animal Center, Hangzhou Normal University. The subject number is No. 167 and the ethics number is No. 302. Female BALB/c mice (6–8 weeks old, 16–24 g) were obtained from Shanghai Slake Experimental Animal Co., Ltd. (China). The 4T1 tumor‐bearing BALB/c mice were established using the method mentioned above. Subsequently, the tumor‐bearing mice were used for antitumor treatment until the tumor volume reached ≈70 mm^3^. The relative tumor volumes were calculated as Vt/V0 (V0 is the tumor volume on the 0th day. 4T1 tumor‐bearing mice were randomly divided into five groups (three mice per group) and treated using different formula through tail intravenous injection: PBS + Light (G1), ZnNCs (G2), DicTBS‐ZnNCs (G3), DicTBS‐ZnNCs + Light (G4), Cyano@DicTBS‐ZnNCs + Light (G5). After 16 h of injection, laser groups were exposed to a laser (300 mW cm^−2^, 660 nm) for 10 min. Each group underwent the procedure every other day three times in the first few days. The tumor size and body weight were measured and recorded every other day using digital calipers and an electronic balance. After the 14th day of treatment, the mice were sacrificed, and their tumor tissues were obtained for photographing. Major organs (hearts, lungs, livers, spleens, and kidneys) were harvested for further histological analysis, and tumors were observed for H&E, TUNEL, and Ki67 staining. Mice survival was independently assessed for a period of 40 days.

### Vivo Transcriptomics

To study the infiltration of immune cells and macrophages into tumor sites after treatment, fresh tumor tissue was collected 10 days after treatment digested into cells using a homogenizer, and filtered through a membrane to obtain a single‐cell suspension. Next, count the cells and prepare cell suspensions of equal cell concentrations for each group. Subsequently, the tumor cell suspensions were stained with fluorescently labeled antibodies according to the manufacturer's instructions. Among them, the detection of macrophages and dendritic cells requires blocking with CD16/32 antibodies in a dark environment at 4 °C for 10 min. The stained cells were then evaluated using a FACSCanto II instrument (BD Biosciences). The instrument parameters are set to collect 5 × 10^5^ cells per group.

To further evaluate the immune response, tumors from the PBS and the Cyano@DicTBS‐ZnNCs + L (660 nm, 300 mW cm^−2^, 10 min) were collected for transcriptomic analysis. Subsequently, the mice were sacrificed, and tumor tissues were collected and frozen in liquid nitrogen for gene sequencing (OE Biotech, Shanghai, China). The heatmap was generated using the platform at OE Biotech, which is used for data analysis and visualization.

### Statistical Analysis

The data were analyzed using the GraphPad Prism. All the data are expressed as the mean ± standard deviation (SD). A one‐way analysis of variance (ANOVA) helped determine statistically significant differences (^*^ *p* < 0.05, ^**^ *p* < 0.01, and ^***^ *p* < 0.001).

## Conflict of Interest

The authors declare no conflict of interest.

## Supporting information



Supporting Information

## Data Availability

The data that support the findings of this study are available in the supplementary material of this article.
