# Supplementary material for: Living Biotherapeutics Using Nanoparticles‐Armed Cyanobacteria for Boosting Photodynamic‐Immunotherapy of Cancer
Source: Adv Sci (Weinh). 2025 May 8;12(27):2502746. doi: 10.1002/advs.202502746 (PMC12279180; doi:10.1002/advs.202502746)
Supplement: Supplementary file 1 — Supporting Information [file ADVS-12-2502746-s001.docx]

Supporting Information

Living Biotherapeutics using Nanoparticles-Armed Cyanobacteria for Boosting Photodynamic-Immunotherapy of Cancer

*Zhengwei Xu ^a,b^, Mingsong Zang ^a^, Hui Li ^a^, Ruizhen Tian ^a^, Zherui Zhang ^a^, Wang Liu ^a^, Fei Xiao ^a^, Xuesha Yan ^a^, Yan Zhu ^a^, Canhong Zhu ^a^, Jiayun Xu ^a^, Yu Shuangjiang ^a^, Tingting Wang ^a *^, Hongcheng Sun ^a *^ and Junqiu Liu ^a,b, *^*

^a^ College of Material, Chemistry and Chemical Engineering, Key Laboratory of Organosilicon Chemistry and Material Technology, Ministry of Education, Hangzhou Normal University, Hangzhou, 311121, P. R. China

^b^ College of Life and Environmental Sciences, Hangzhou Normal University, Hangzhou Normal University, Hangzhou, 311121, P. R. China

**Experimental Section**

**1. Chemicals and Materials**

The experimental reagents and materials used in this chapter were obtained through commercial purchase and were not subjected to further purification. All solvents, including N,N-dimethylformamide (DMF), diethyl ether, acetonitrile, propionic acid, methanol, and dichloromethane (DCM), were procured from Beijing Chemical Works. Prior to use, N,N-dimethylformamide (DMF) and acetonitrile were treated with anhydrous calcium hydride to ensure moisture-free conditions. All reagents, such as pyrrole, 4-pyridinecarboxaldehyde, cisplatin, triethylamine (TEA), dicoumarol, tert-butyldimethylchlorosilane (TBSCl), hydrogen peroxide (H_2_O_2_), 4-dimethylaminopyridine (DMAP), dicyclohexylcarbodiimide (DCC), and 5,5-dimethyl-1-pyrroline N-oxide (DMPO), were purchased from Anrui Chemical Company. Agar was sourced from Shenggong Biotech (Shanghai) Co., Ltd. BG-11 medium and cyanobacteria (Synechococcus sp. FACHB-805) were acquired from the Freshwater Algal Germplasm Bank of the Chinese Academy of Sciences. 3-(4,5-dimethylthiazol-2-yl)-2,5-diphenyltetrazolium bromide (MTT) was obtained from Dalian Meilun Biotechnology Co., Ltd. DMEM high glucose medium, fetal bovine serum (FBS), a mixture of penicillin and streptomycin, trypsin digestion solution, cell culture dishes, 96-well plates, DCFH-DA reactive oxygen species assay kit, 4% paraformaldehyde fixative, Hoechst 33342 staining kit, Annexin V-FITC/PI apoptosis detection kit, and Calcein-AM/PI live-dead cell staining kit were all purchased from Shenggong Bioengineering (Shanghai) Co., Ltd. The water used in the experiments of this chapter was Milli-Q water obtained through double distillation, with a resistivity of 18.2 MΩ•cm.

**2. Characterizations**

Nuclear Magnetic Resonance (NMR) Spectrometer (AVANCEIII 500), Liquid Chromatography-Mass Spectrometer (LC-MS, Agilent 1290-microTOD-Q II), and Matrix-Assisted Laser Desorption/Ionization Time-of-Flight Mass Spectrometer (Autoflex speed TOF/TOF) are manufactured by Bruker Corporation, Germany. Scanning Electron Microscope (JSM6700F), Transmission Electron Microscope (JEM-2100F), and Electron Spin Resonance Spectrometer (JES-FA 200) are produced by Japan Electronics Corporation. UV-Vis Spectrophotometer (3100 UV-Vis) and Fluorescence Spectrophotometer (RF-5301-PC) are produced by Shimadzu Corporation, Japan. Laser Confocal Inverted Microscope (LSM 710) is produced by Carl Zeiss, Germany. Flow Cytometer (BD Accuri C6, USA) is produced by Becton, Dickinson and Company (BD), USA. Nanoparticle Size Analyzer (ZS 90) is produced by Malvern Corporation, UK. Microplate Reader is produced by Nanjing De Tie Laboratory Equipment Co., Ltd. The 660-nanometer Near-Infrared Laser (FC660LH-300 mW-FC) is produced by Changchun New Industries Optoelectronics Tech Co., Ltd, China. The Light Exposure Cultivation Chamber (MGC-250BP-2) is produced by Shanghai Yiheng Technical Co., Ltd. The Dissolved Oxygen Meter (DO-957) is produced by Shanghai INESA Scientific Instrument Co., Ltd.


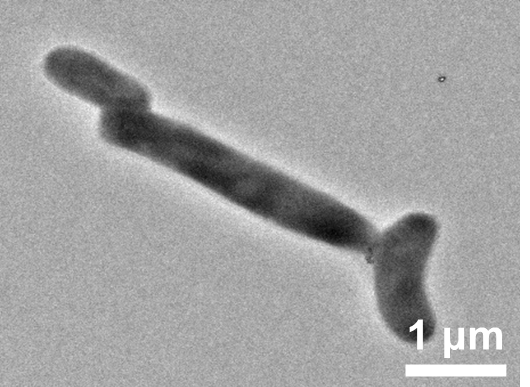


**Figure S1.** TEM image of Cyanobacteria.


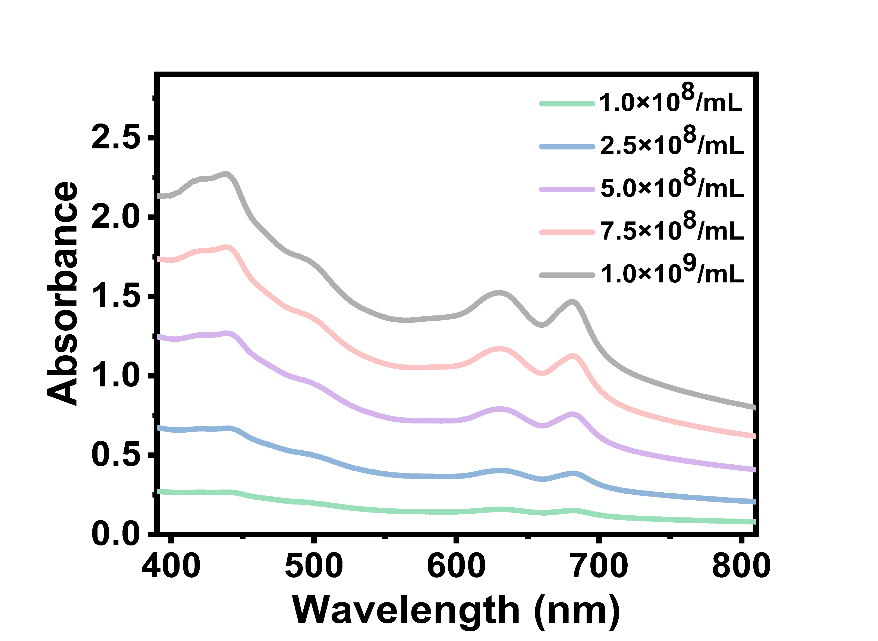


**Figure S2.** UV-vis absorbance spectra of Cyanobacteria.


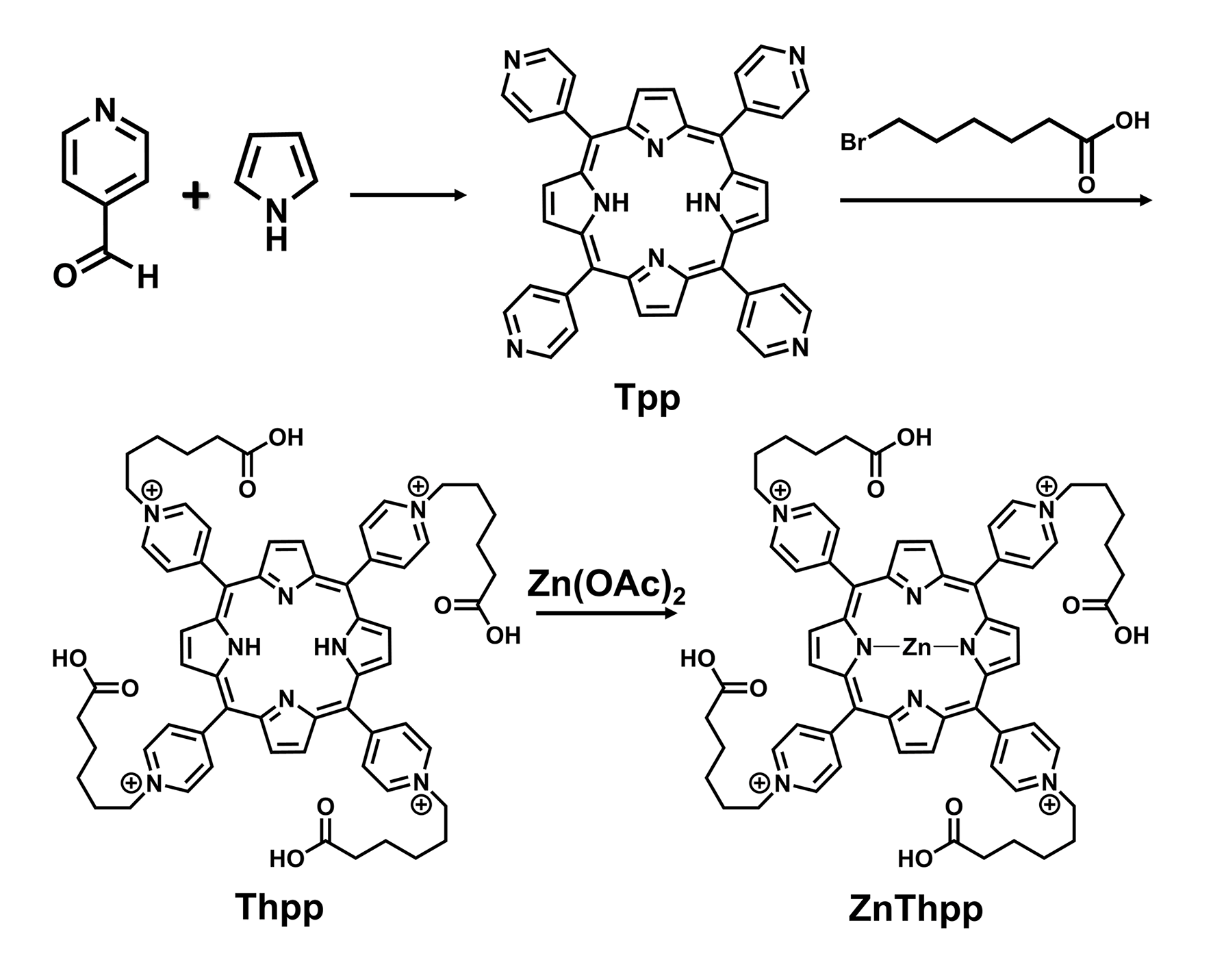


**Scheme S3**. Design and synthetic route of ZnThpp.


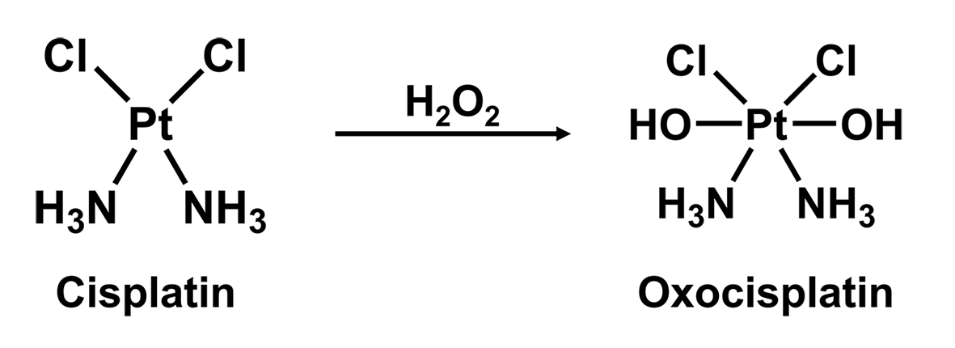


**Figure S4.** Synthetic route of Oxocisplatin.


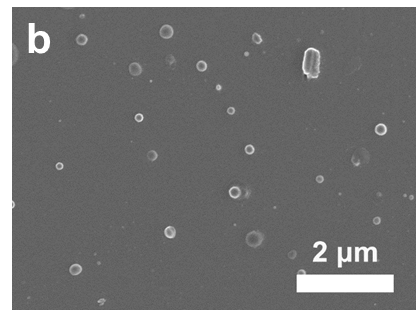


**Figure S5.** SEM images of ZnNCs.


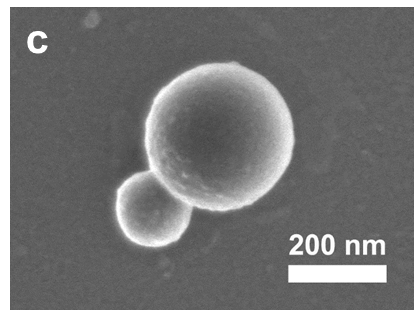


**Figure S6.** SEM images of Dic-ZnNCs.


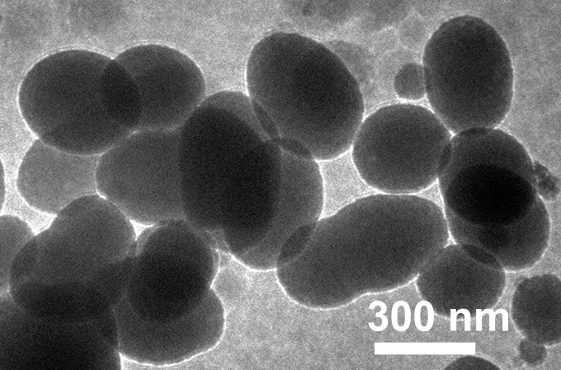


**Figure S7.** TEM image of DicTBS-ZnNCs.


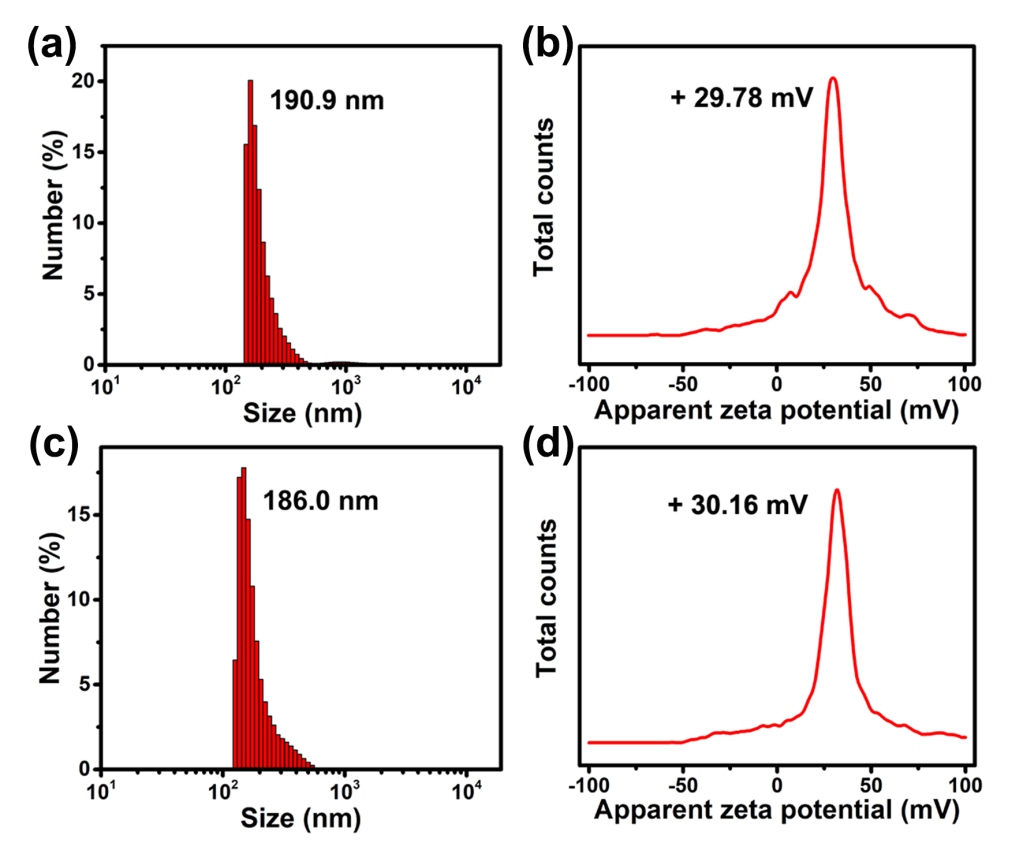


**Figure S8.** (a) Size distribution by number and (b) zeta potential of ZnNCs. (c) Size distribution by number and (d) zeta potential of DicTBS-ZnNCs.


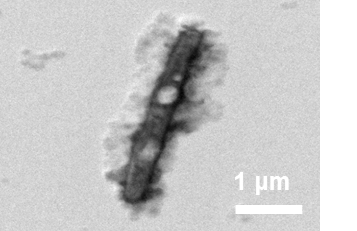


**Figure S9.** TEM image of Cyano@DicTBS-ZnNCs.

The 2 mg bio-hybrid was treated under acidic conditions to completely disassemble and release ZnThpp. The zinc (Zn) concentration in the resulting 5 mL solution was measured to be 2.79 ppm, corresponding to a total of 13.95 μg of Zn in the system. Therefore, the ZnThpp content in the 2 mg nanoparticle capsule was calculated to be 1142.06 μg, resulting in a loading efficiency of 12.18%.


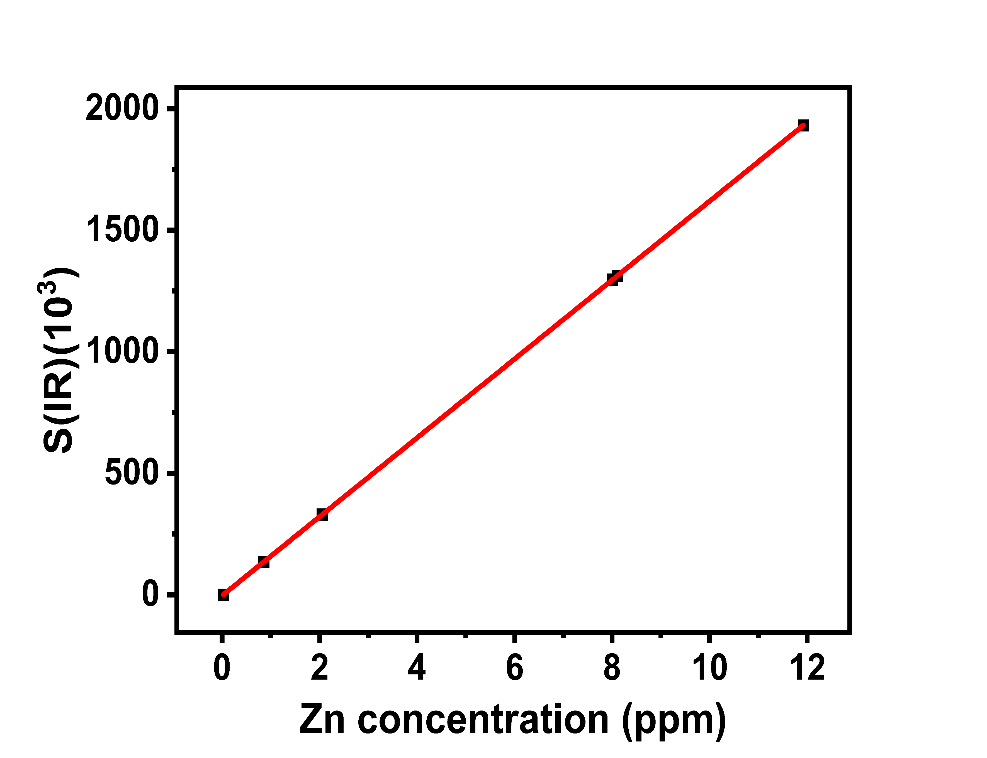


**Figure S10.** The standard curve of Zn elements from ICP-MS.


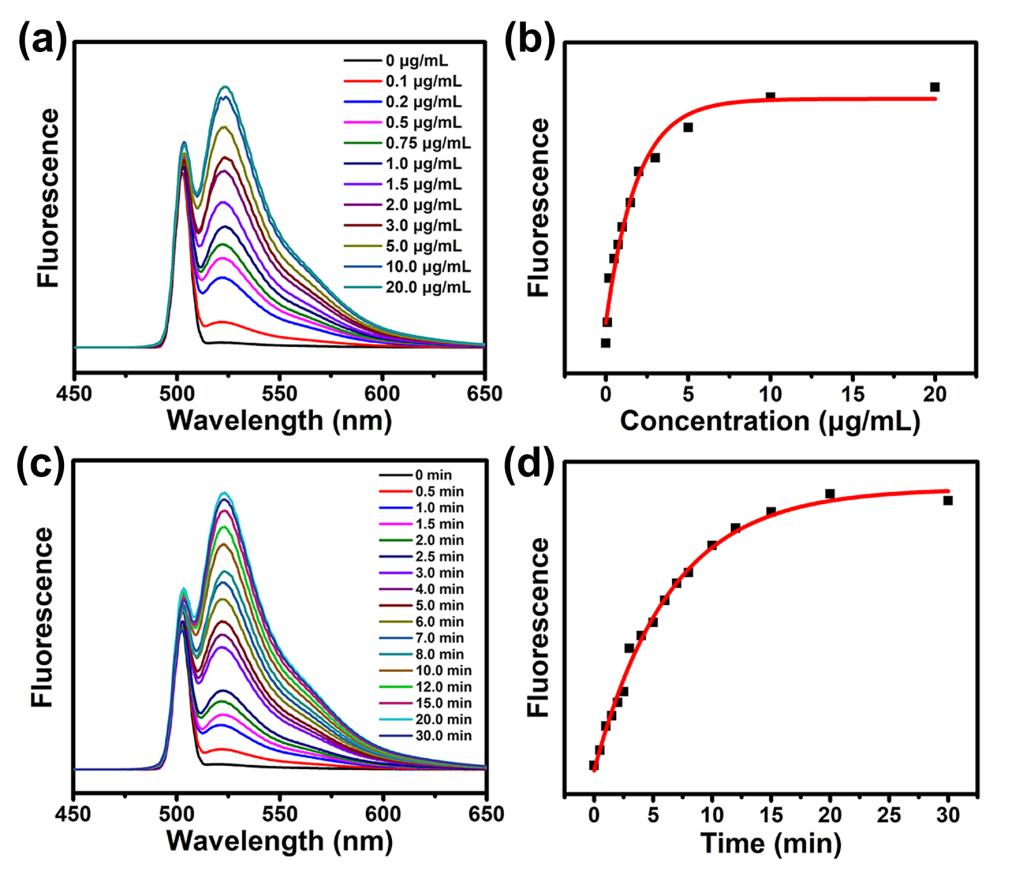


**Figure S11.** (a) Fluorescence spectrum and (b) fluorescence intensity at 524 nm of DCFH treated with different concentrations of ZnNCs under 660 nm light (DCFH concentration: 4 μM; 660 nm light, 300 mW cm ^-2^,10 min). (c) Fluorescence spectrum and (d) fluorescence intensity at 524 nm of DCFH treated with ZnNCs under different times of 660 nm light (DCFH concentration: 4 μM; 660 nm light, 300 mW cm^-2^).


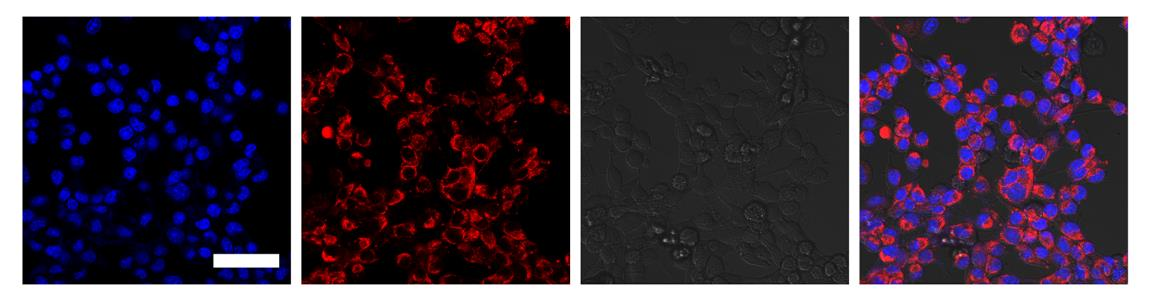


**Figure S12.** CLSM images of 4T1 cells treated with free ZnNCs (Scale bar, 50 μm). Cell nuclei stained with Hoechst 33342 (blue channel).


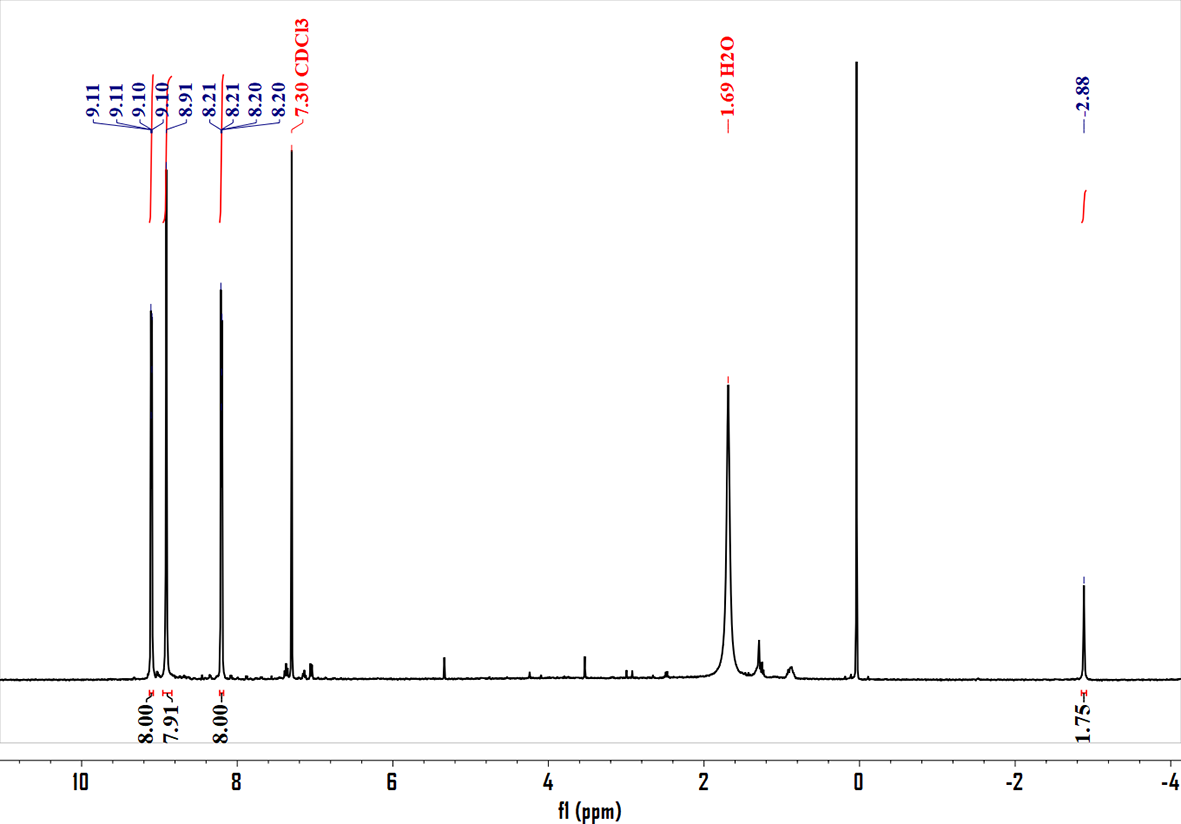


**Figure S13.** ^1^H NMR (Chloroform-d) spectrum of synthesized Tpp.


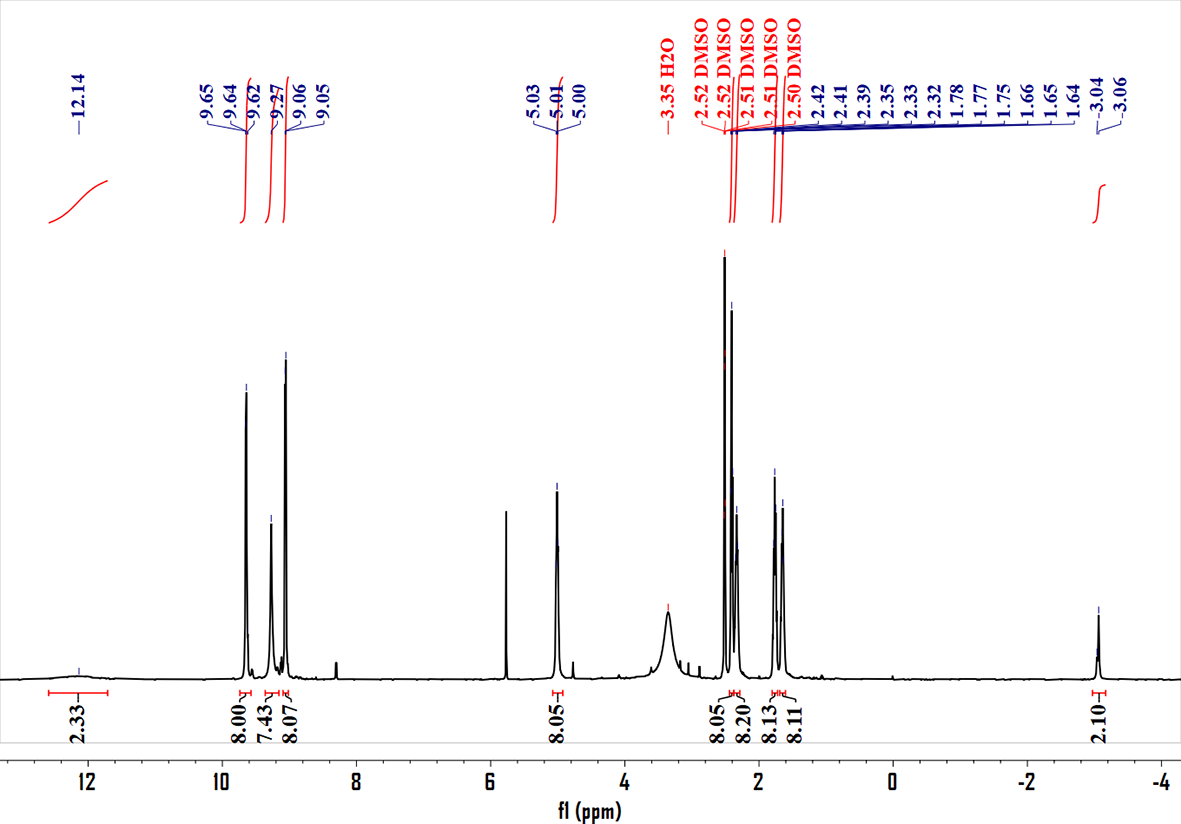


**Figure S14.** ^1^H NMR (DMSO-d6) spectrum of synthesized Thpp.


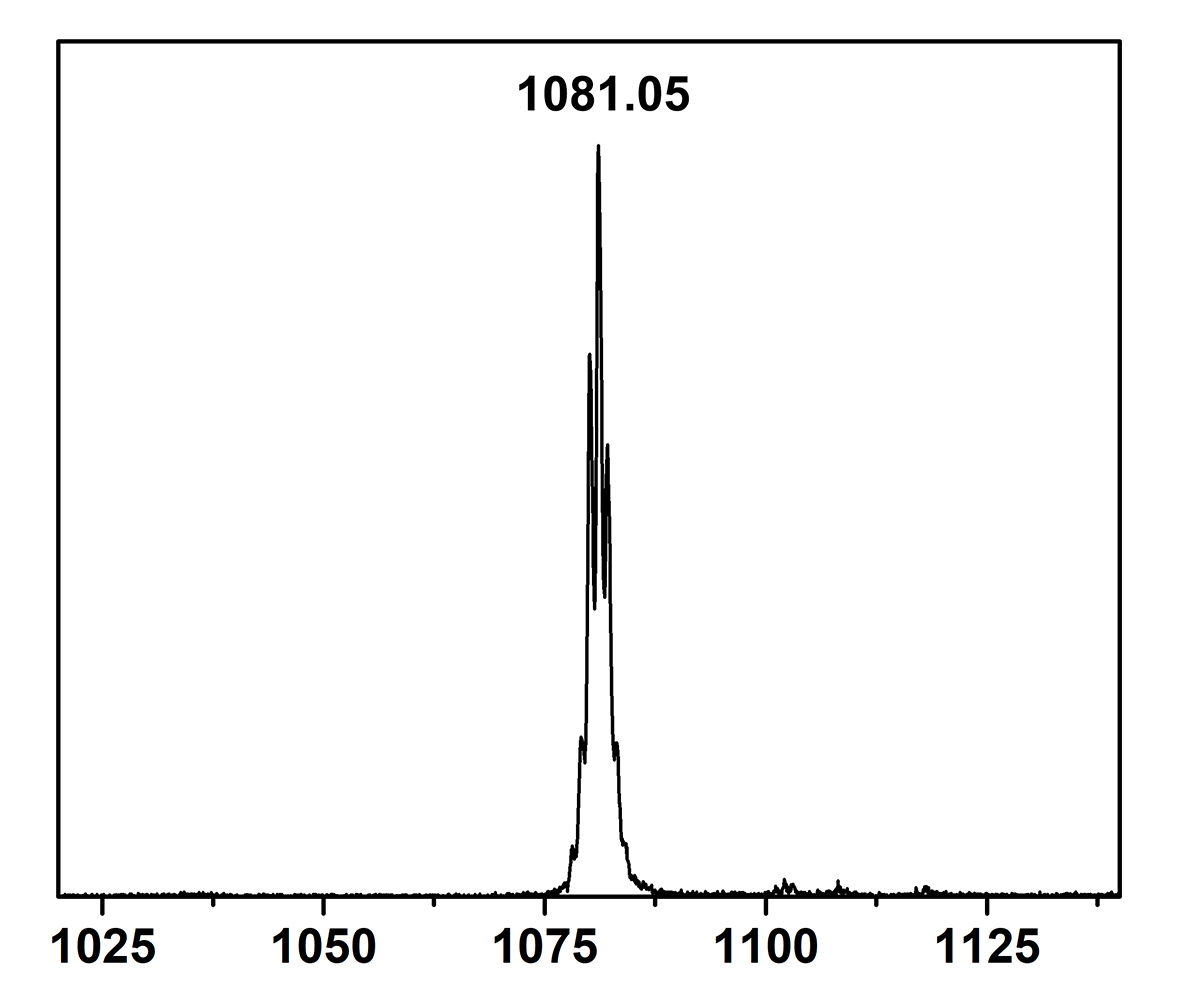


**Figure S15.** MALDI -TOF mass spectrum of Thpp.


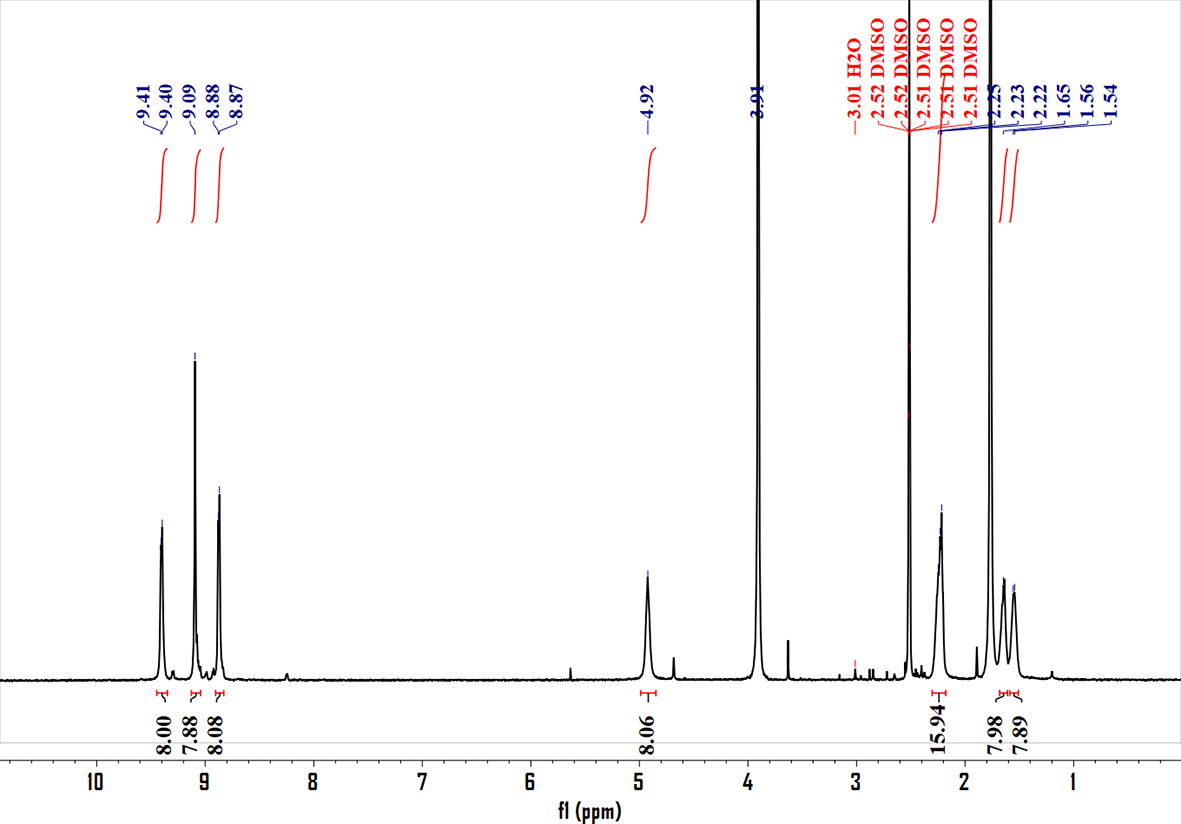


**Figure S16.** 1H-NMR (DMSO-d6) spectrum of ZnThpp.


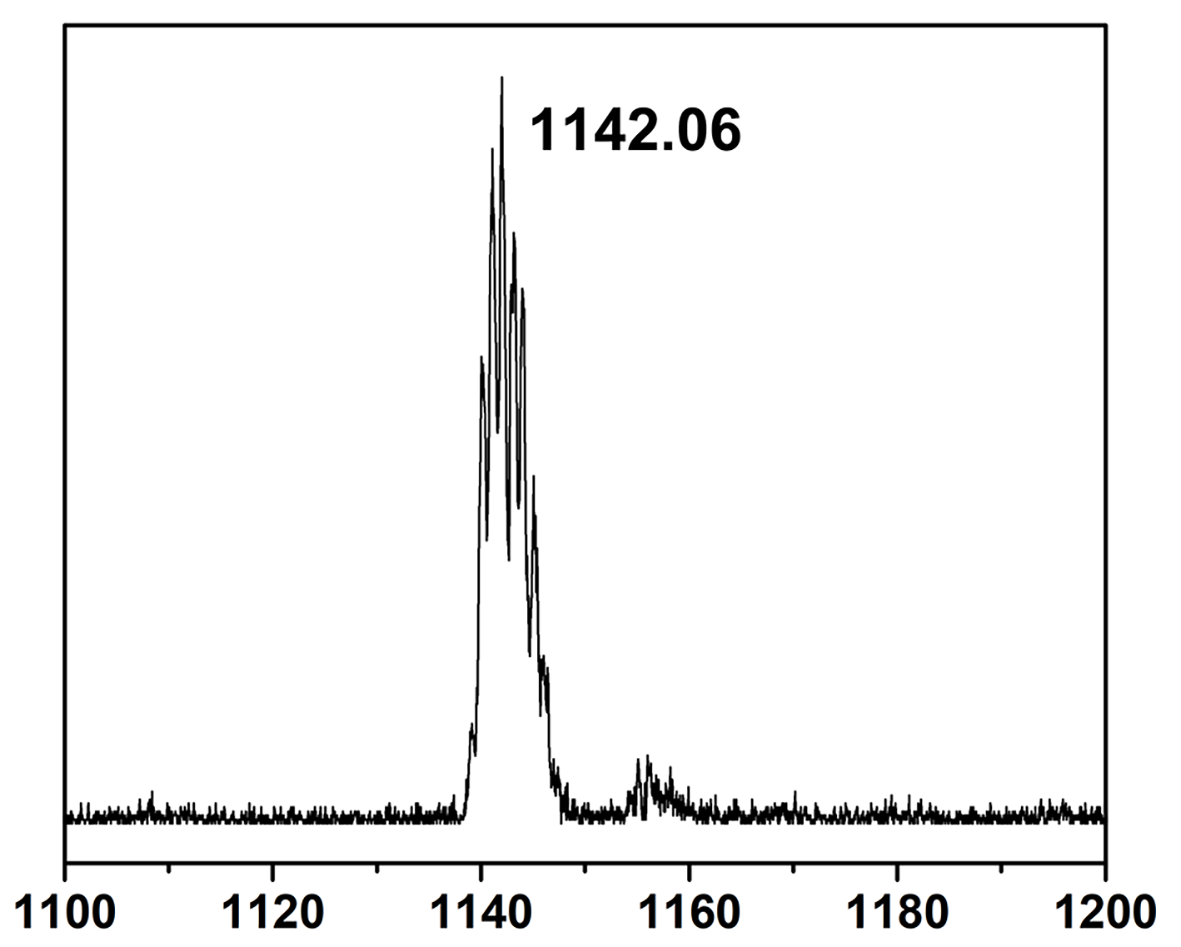


**Figure S17.** MALDI -TOF mass spectrum of ZnThpp.


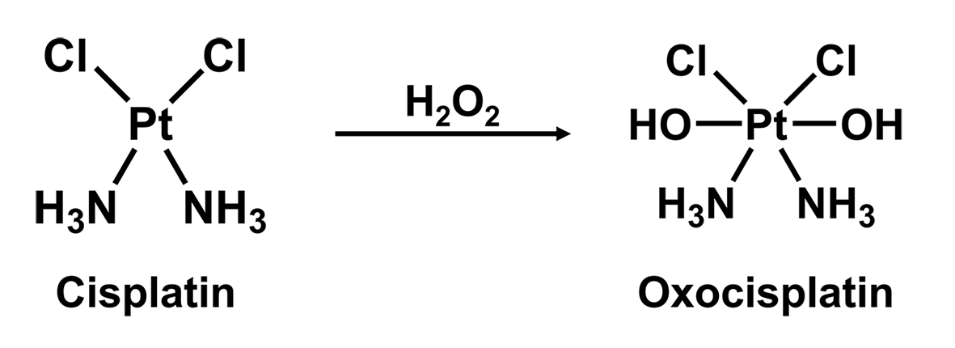


**Figure S18.** Synthetic route of Oxocisplatin.


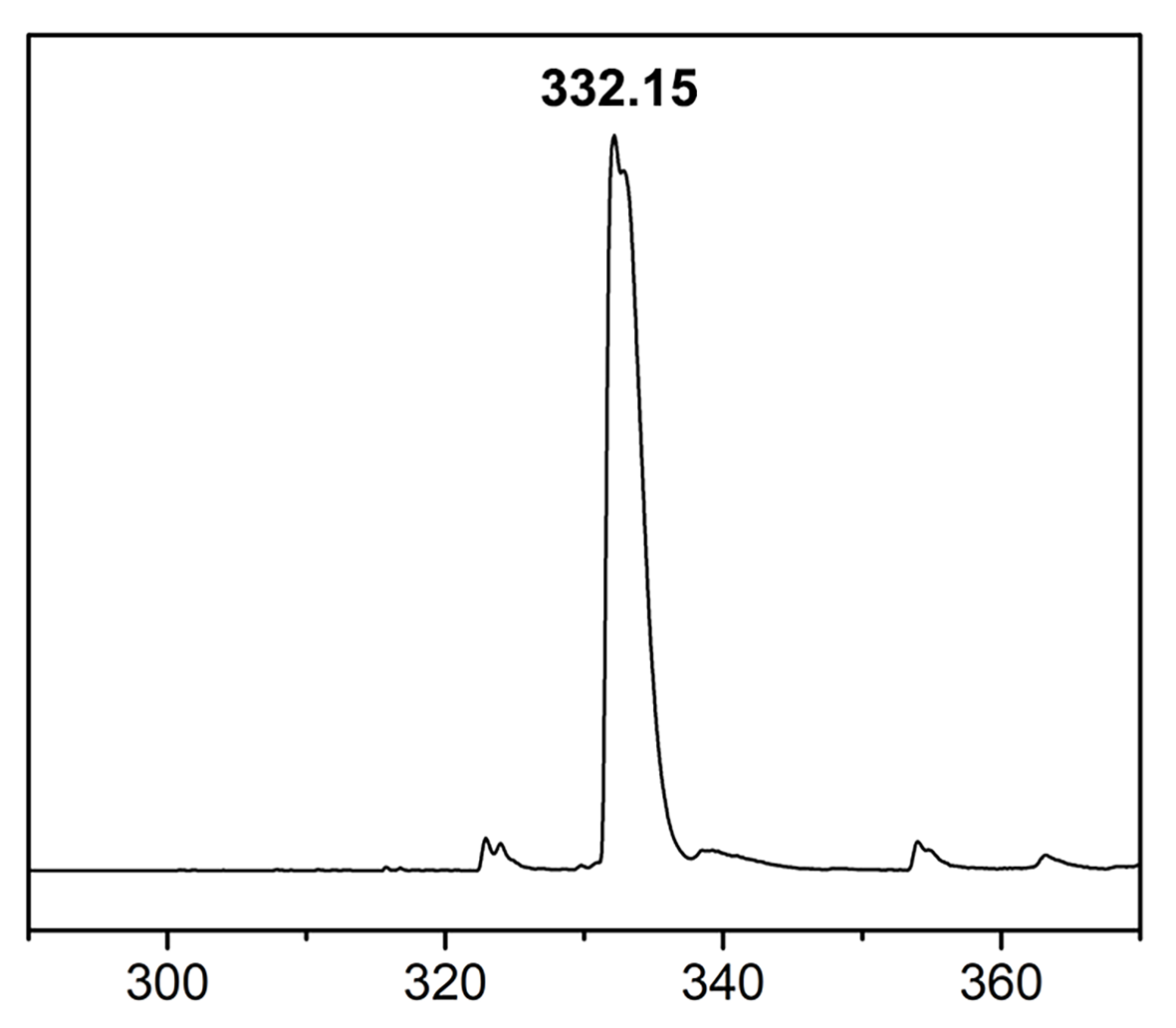


**Figure S19.** MALDI-TOF mass spectrum of Oxocisplatin.


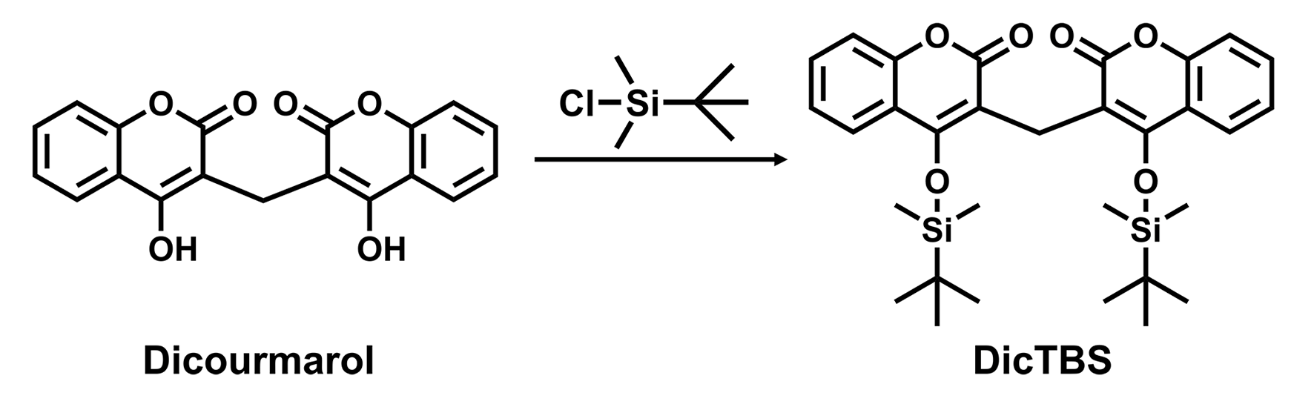


**Figure S20.** Synthetic route of DicTBS.


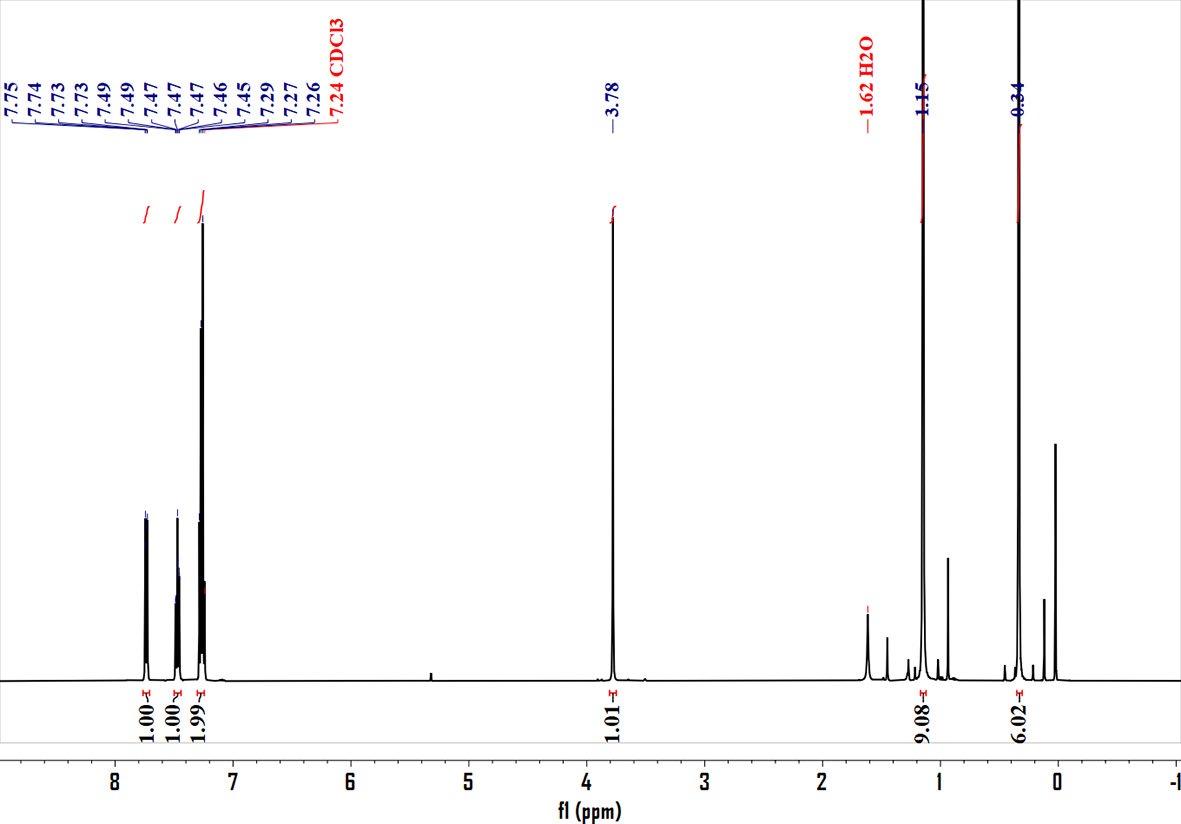


**Figure S21.** ^1^H NMR (Chloroform-d) spectrum of synthesized DicTBS.


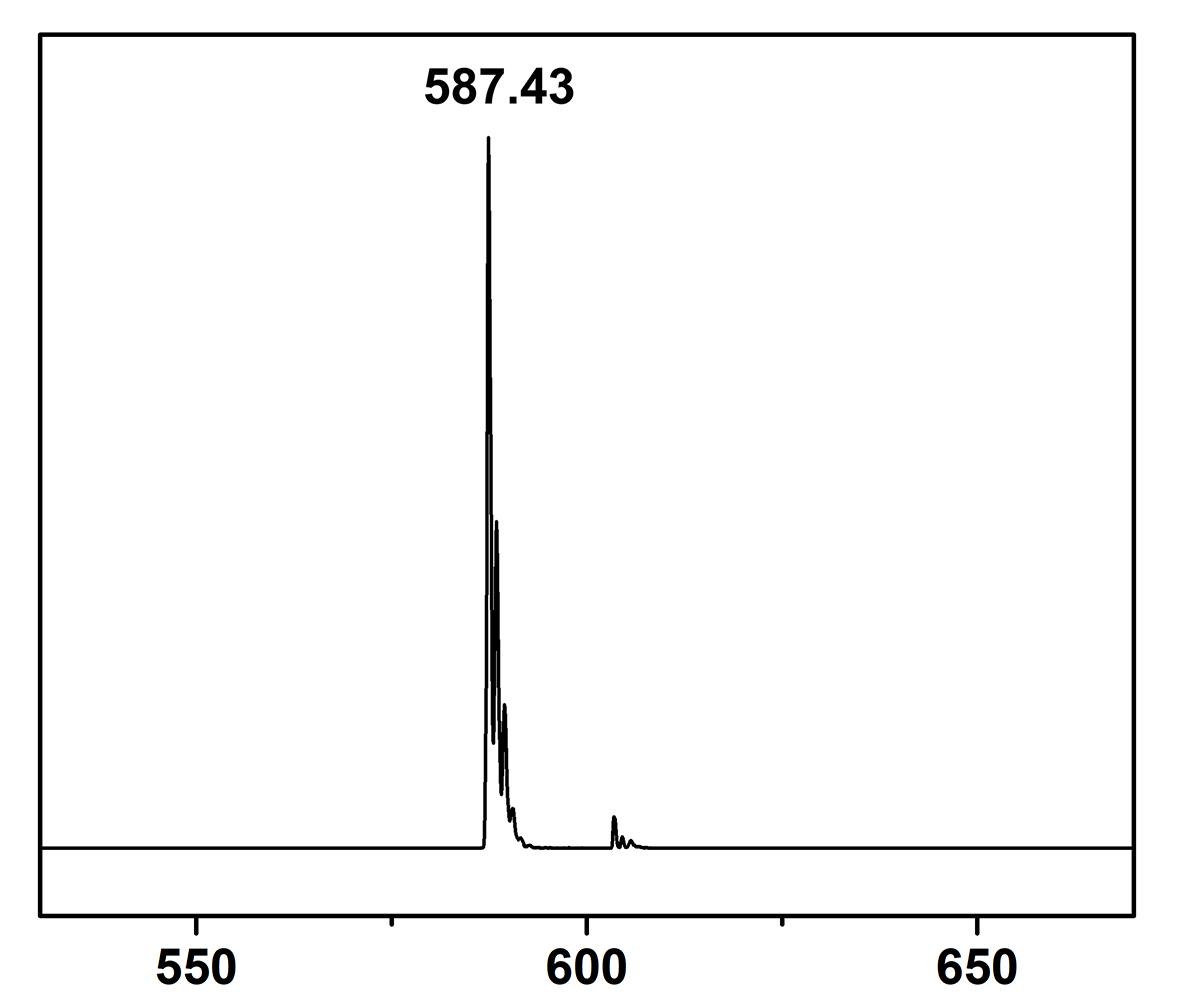


**Figure S22.** MALDI-TOF mass spectrum of DicTBS.

**The Loading Percentage:** The Oxocisplatin and ZnThpp in the Cyano@DicTBS-ZnNCs were measured by ICP-MS respectively, using their own standard curves. The loading percentage (LP) of each component in Cyano@DicTBS-ZnNCs were determined as follows

LP (i, %) = m_i_ (in Cyano@DicTBS-ZnNCs ) **/** m (Cyano@DicTBS-ZnNCs ) × 100%

where mi and m are the mass values of testing component in Cyano@DicTBS-ZnNCs , initially added component when making Cyano@DicTBS-ZnNCs , and total Cyano@DicTBS-ZnNCs , respectively. As detected, the LP values of Oxocisplatin and ZnThpp were measured to be 19.29% and 12.18%, respectively.

The 2 mg bio-hybrid was treated under acidic conditions to fully disassemble and release oxaliplatin. The platinum (Pt) concentration in the resulting 5 mL solution was measured to be 45.32 ppm, corresponding to a total of 226.6 μg of Pt in the system. Thus, the oxaliplatin content in the 2 mg nanoparticle capsule was calculated to be 385.81 μg, resulting in a platinum loading efficiency of 19.29%.


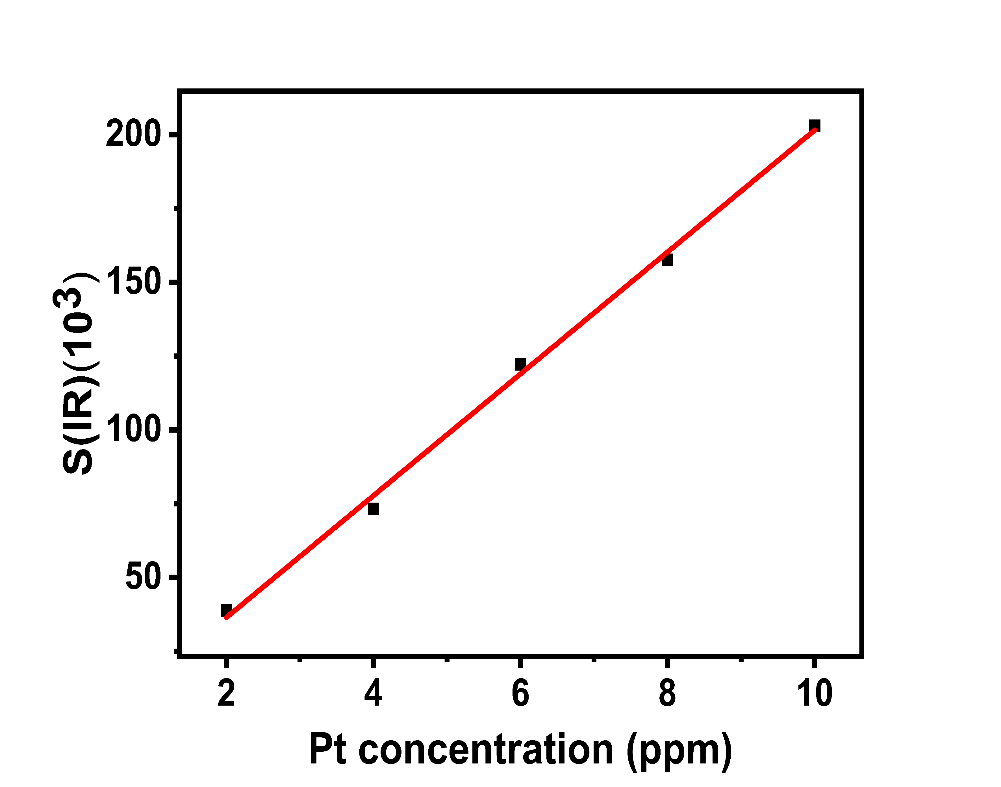


**Figure S23.** The standard curve of Pt elements from ICP-MS.

**The release Percentage:** It was found that the Cyano@DicTBS-ZnNCs showed pH-dependent cargo release proﬁle and more than 70% cargo was released under the simulated tumor surrounding (pH 5.0) after 24 h, while only ≈20 % for normal cells (pH = 7.4) (Figure S24). In addition, the GSH responsive release of cisplatin from this Cyano@DicTBS-ZnNCs was investigated by the ICP-MS. It was found that the cumulative Pt contents released from Cyano@DicTBS-ZnNCs were nearly 64.5 % and 30.7 % when incubated with 1 mM and 10 mM GSH (pH = 7.4), responsively, while that was only 6.4 % in PBS buﬀer (pH = 7.4).


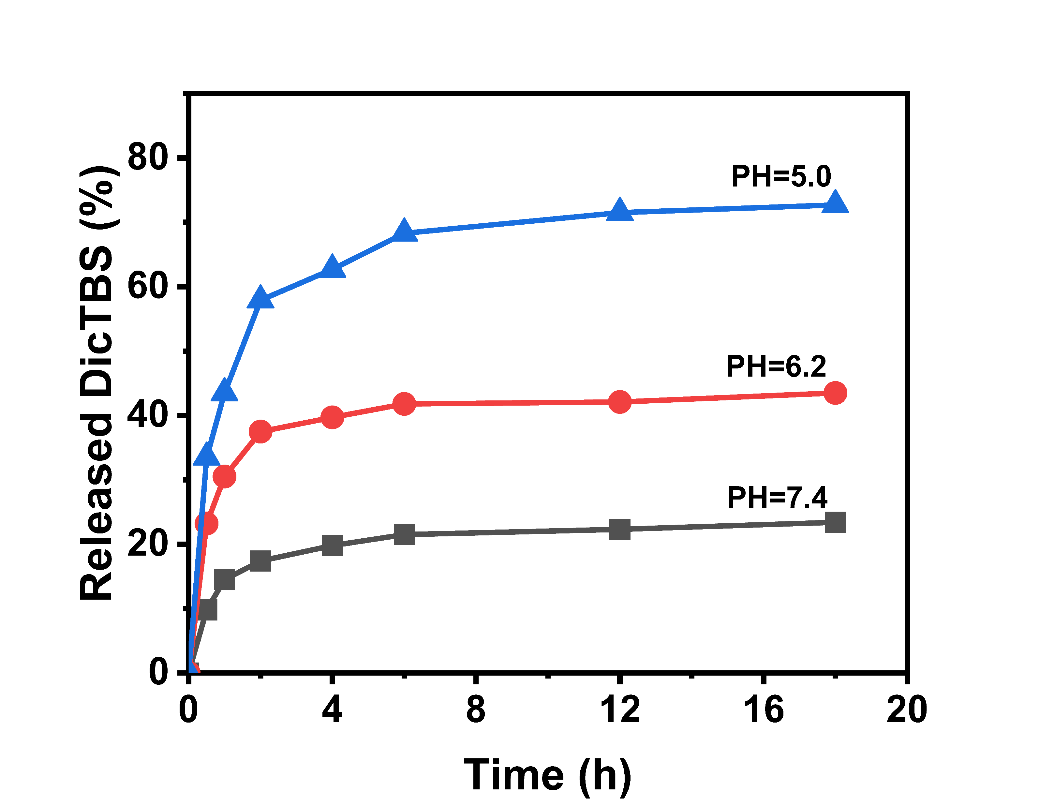


**Figure S24.** DicTBS release proﬁles of Cyano@DicTBS-ZnNCs in PBS under pH 7.4, 6.2, and 5.0 for 20 h.


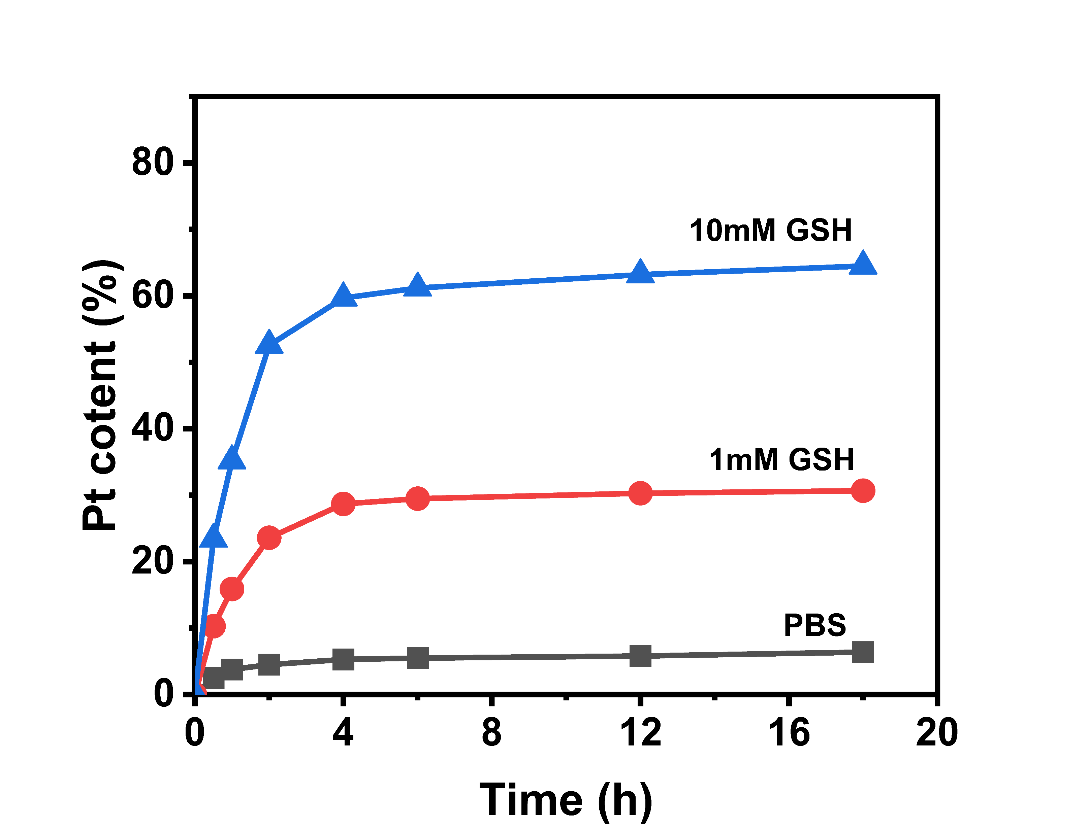


**Figure S25.** Pt element release proﬁles of Cyano@DicTBS-ZnNCs in PBS (pH 7.4) with diﬀerent amount of GSH (0 × 10^−3^, 1.0 × 10^−3^, 10 × 10^−3^ M).

**Determination of dissolved oxygen within the tumor volume:** To estimate the oxygen concentration in tumor tissue, the oxygen content in mouse tumor tissue was measured using an oxygen meter (oxygen electrode method). The electrode probe was inserted into the tissue to measure the local oxygen concentration.


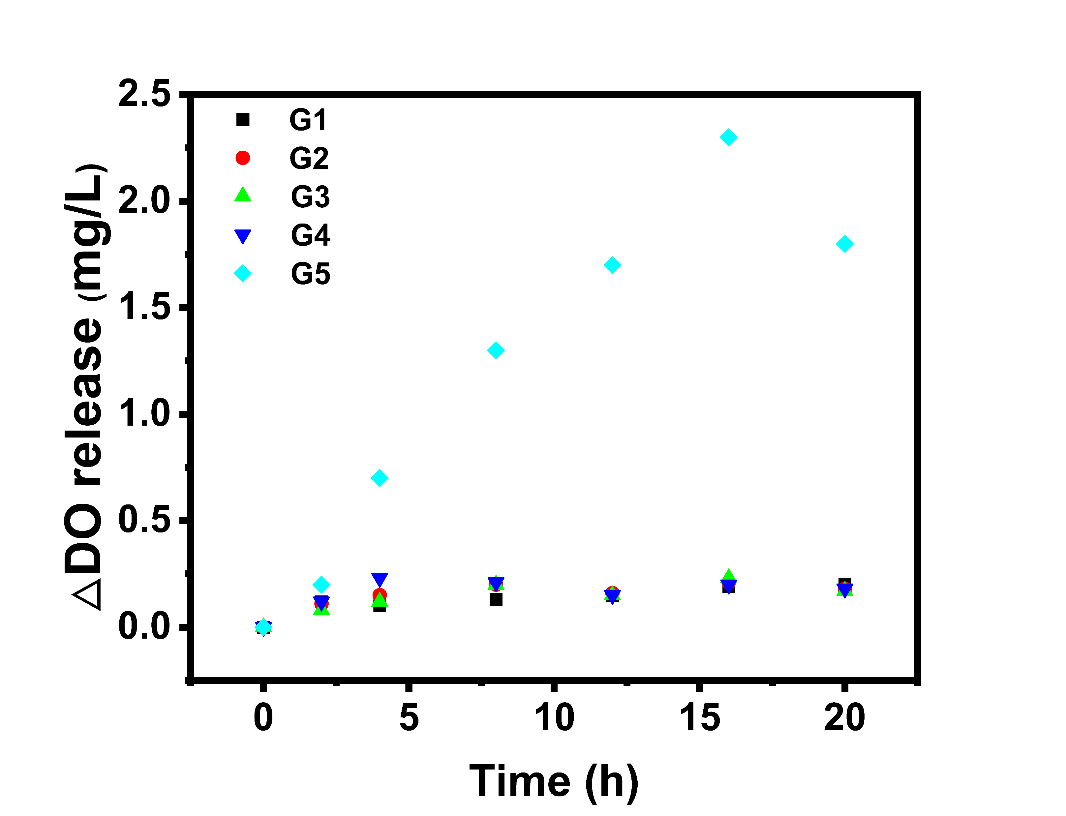


**Figure S26.** Oxygen concentration in tumor tissues at different time points.


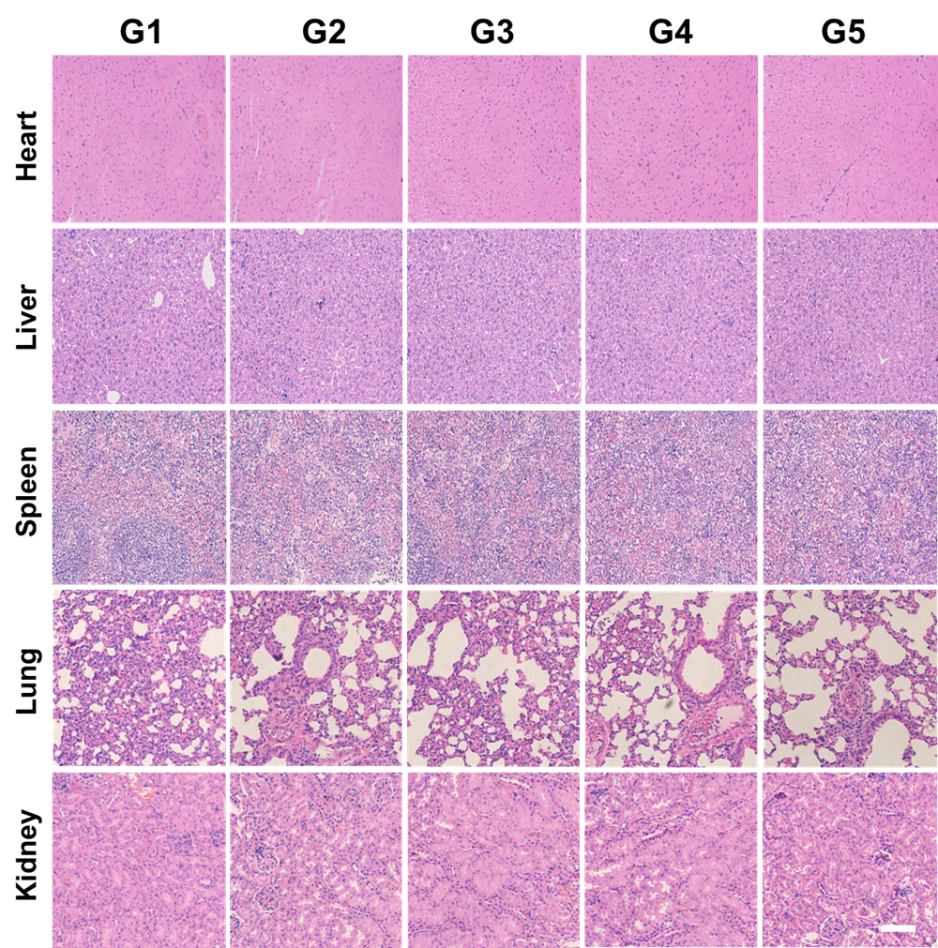


**Figure S27.** H&E staining images of major mice organs (heart, liver, spleen, lung, kidney) after injecting PBS + L (G1), ZnNCs (G2), DicTBS-ZnNCs (G3), DicTBS-ZnNCs +L (G4), and Cyano@DicTBS-ZnNCs + L (G5). The scale bar is 100 μm.


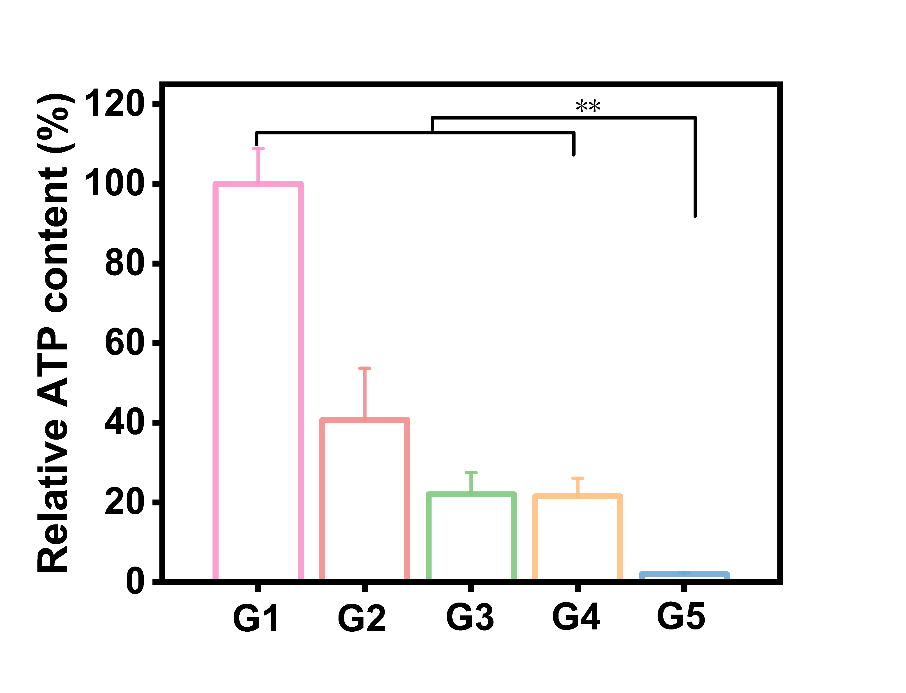


**Figure S28.** ATP levels 4T1 cells a fter various treatments for 20 h (n = 3).
